# Supplementary material for: Temporal trends in incidence of atrial fibrillation in primary care records: a population-based cohort study
Source: BMJ Open. 2020 Dec 23;10(12):e042518. doi: 10.1136/bmjopen-2020-042518 (PMC7759967; doi:10.1136/bmjopen-2020-042518)
Supplement: Supplementary data [file bmjopen-2020-042518supp001.pdf]

Appendices

Appendix 1: Number of practices contributing data to CPRD GOLD during study period. Figures are mid-year counts.

	2004	2006	2008	2010	2012	2014	2016	2018
UK practices	680	708	717	703	672	599	448	367
English practices	451	460	462	443	411	335	185	110
Scottish, Welsh and Northern Irish practices	229	248	255	260	261	264	263	257

Appendix 2: List of codes used to define Atrial Fibrillation. The presence of these codes in the patient record, in the clinical and referral tables, was considered as evidence of diagnosis.

Medcode	Readcode	Readterm
1664	G573000	Atrial fibrillation
2212	G573.00	Atrial fibrillation and flutter
1268	G573200	Paroxysmal atrial fibrillation
1757	G573100	Atrial flutter
23437	G573z00	Atrial fibrillation and flutter NOS
96076	G573500	Persistent atrial fibrillation
96277	G573400	Permanent atrial fibrillation
35127	G573300	Non-rheumatic atrial fibrillation

Mendonça SC, *et al. BMJ Open* 2021; 10:e042518. doi: 10.1136/bmjopen-2020-042518

Appendix 3: Flowchart for cohort - incident cases of AF from English practices.

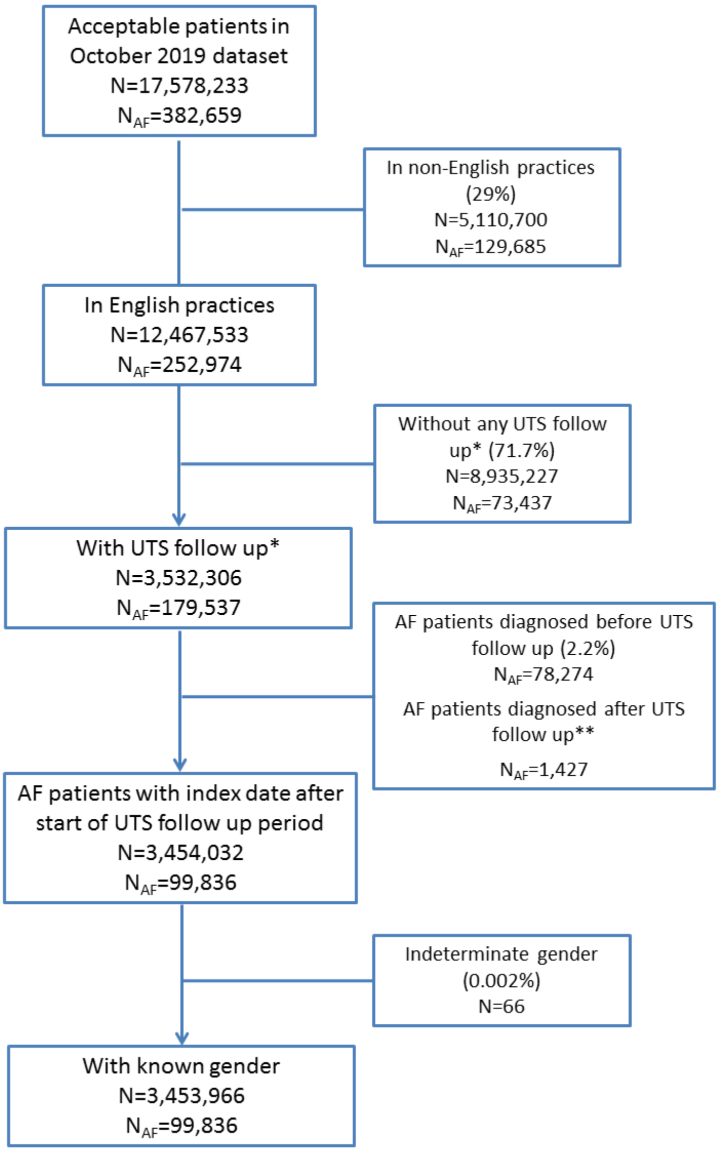

Notes: Each step shows the total number of patients in the denominator (N) and the sub-sample with AF in the numerator (N_{AF}). *"Up-to-standard" (UTS) follow up as defined in the methods section. ** N patients to be excluded from numerator but will contribute to denominator.

Appendix 4: Observed and modelled incidence rates (per 1,000 person-years) for recorded AF during the period 2004-2018– stratified by calendar year and gender.

Observed rates						
	Both genders		Women		Men	
Year	Rate	95% CI	Rate	95% CI	Rate	95% CI
2004	4.46	4.36-4.56	4.23	4.10-4.37	4.71	4.57-4.86
2005	4.31	4.21-4.40	4.03	3.90-4.16	4.61	4.46-4.75
2006	4.46	4.36-4.56	4.17	4.05-4.31	4.77	4.62-4.91
2007	4.31	4.22-4.41	3.95	3.83-4.08	4.69	4.55-4.84
2008	4.37	4.28-4.47	4.11	3.99-4.24	4.64	4.51-4.79
2009	4.32	4.23-4.42	3.98	3.85-4.10	4.69	4.55-4.84
2010	4.24	4.15-4.34	3.81	3.68-3.94	4.71	4.57-4.86
2011	4.49	4.39-4.59	4.07	3.94-4.21	4.93	4.78-5.08
2012	4.77	4.67-4.87	4.31	4.18-4.45	5.26	5.10-5.41
2013	4.69	4.58-4.80	4.17	4.04-4.32	5.24	5.08-5.41
2014	4.80	4.69-4.92	4.21	4.06-4.36	5.44	5.27-5.62
2015	5.07	4.94-5.20	4.55	4.38-4.73	5.62	5.42-5.82
2016	4.89	4.74-5.05	4.44	4.24-4.65	5.38	5.15-5.61
2017	4.77	4.60-4.95	4.02	3.81-4.25	5.56	5.30-5.84
2018	4.58	4.40-4.78	3.86	3.63-4.11	5.34	5.06-5.64
Modelled rates(1)						
	Both genders		Women		Men	
	Rate	95% CI	Rate	95% CI	Rate	95% CI
2004	4.44	4.34-4.54	4.14	4.01-4.28	4.75	4.60-4.90
2005	4.30	4.21-4.40	3.98	3.85-4.11	4.66	4.51-4.80
2006	4.47	4.38-4.57	4.14	4.01-4.27	4.84	4.69-4.98
2007	4.33	4.23-4.43	3.93	3.80-4.05	4.76	4.62-4.91
2008	4.40	4.30-4.49	4.11	3.98-4.24	4.71	4.56-4.85
2009	4.35	4.26-4.45	3.99	3.86-4.12	4.74	4.60-4.89
2010	4.27	4.18-4.37	3.83	3.71-3.96	4.75	4.60-4.90
2011	4.50	4.40-4.60	4.10	3.97-4.23	4.93	4.78-5.09
2012	4.75	4.65-4.86	4.33	4.19-4.47	5.21	5.05-5.37
2013	4.65	4.55-4.76	4.19	4.05-4.33	5.14	4.98-5.31
2014	4.72	4.61-4.84	4.19	4.04-4.34	5.29	5.11-5.46
2015	4.97	4.84-5.11	4.54	4.37-4.72	5.44	5.24-5.64
2016	4.84	4.69-5.00	4.48	4.28-4.69	5.23	5.00-5.47
2017	4.86	4.69-5.04	4.21	3.99-4.45	5.55	5.29-5.83
2018	4.66	4.47-4.85	4.05	3.81-4.30	5.31	5.02-5.60

(1) Modelled rates take into account the effect of changes in age-gender case mix over time.

Appendix 5: Observed and modelled incidence rates (per 1,000 person-years) for recorded AF during the period 2004-2018 – stratified by calendar year, age and gender.

Both genders – Observed rates										
	40-54		55-64		65-74		75-84		85+	
Year	Rate	95% CI	Rate	95% CI	Rate	95% CI	Rate	95% CI	Rate	95% CI
2004	0.56	0.51-0.62	2.33	2.19-2.48	6.99	6.69-7.29	15.1	14.6-15.7	20.0	19.0-21.2
2005	0.62	0.57-0.68	2.21	2.08-2.35	6.83	6.54-7.13	14.6	14.0-15.1	19.1	18.0-20.1
2006	0.54	0.49-0.59	2.43	2.29-2.58	6.98	6.69-7.28	15.4	14.9-16.0	20.0	19.0-21.1
2007	0.55	0.50-0.60	2.30	2.16-2.44	6.70	6.42-7.00	14.4	13.9-15.0	21.0	20.0-22.1
2008	0.54	0.49-0.59	2.41	2.28-2.56	6.86	6.57-7.15	14.8	14.3-15.4	20.4	19.4-21.5
2009	0.55	0.50-0.61	2.28	2.14-2.42	6.60	6.32-6.89	14.6	14.0-15.1	21.9	20.9-23.0
2010	0.51	0.46-0.56	2.24	2.11-2.39	6.49	6.21-6.79	14.8	14.3-15.4	20.4	19.4-21.5
2011	0.57	0.52-0.62	2.42	2.27-2.57	6.65	6.37-6.95	15.5	14.9-16.1	21.9	20.9-23.0
2012	0.57	0.52-0.63	2.42	2.27-2.58	7.27	6.97-7.58	16.5	15.9-17.1	22.8	21.7-23.9
2013	0.62	0.56-0.68	2.35	2.19-2.51	6.74	6.45-7.04	16.3	15.7-16.9	23.1	21.9-24.3
2014	0.62	0.56-0.69	2.38	2.21-2.55	6.86	6.55-7.19	16.7	16.0-17.4	23.2	22.0-24.4
2015	0.68	0.61-0.75	2.62	2.43-2.83	6.86	6.51-7.22	17.7	17.0-18.5	24.9	23.5-26.3
2016	0.64	0.56-0.73	2.41	2.20-2.65	7.18	6.76-7.63	16.5	15.6-17.4	24.9	23.2-26.6
2017	0.62	0.53-0.72	2.57	2.32-2.85	7.10	6.62-7.62	16.7	15.7-17.8	24.8	22.9-26.9
2018	0.63	0.54-0.75	2.37	2.11-2.66	6.97	6.45-7.53	15.7	14.7-16.9	23.7	21.7-26.0
Women – Observed rates										
	40-54		55-64		65-74		75-84		85+	
Year	Rate	95% CI	Rate	95% CI	Rate	95% CI	Rate	95% CI	Rate	95% CI
2004	0.293	0.243-0.354	1.52	1.36-1.69	5.61	5.25-5.99	13.9	13.2-14.6	19.1	17.9-20.5
2005	0.369	0.313-0.435	1.40	1.26-1.57	5.38	5.03-5.75	13.1	12.4-13.7	18.6	17.4-19.9
2006	0.319	0.267-0.380	1.64	1.48-1.81	5.70	5.34-6.09	13.6	13.0-14.3	18.7	17.6-20.0
2007	0.314	0.263-0.375	1.52	1.37-1.69	5.00	4.66-5.36	12.7	12.1-13.4	19.7	18.5-21.0
2008	0.293	0.245-0.352	1.61	1.46-1.79	5.45	5.10-5.82	13.5	12.8-14.2	19.7	18.5-21.0
2009	0.286	0.238-0.344	1.42	1.27-1.58	5.04	4.71-5.40	13.0	12.4-13.7	20.9	19.6-22.2
2010	0.283	0.234-0.341	1.50	1.35-1.67	4.87	4.54-5.22	12.8	12.2-13.5	18.4	17.3-19.7
2011	0.298	0.248-0.359	1.70	1.54-1.89	5.18	4.84-5.55	13.3	12.7-14.1	20.3	19.0-21.6
2012	0.306	0.255-0.368	1.49	1.33-1.67	5.56	5.21-5.94	14.6	13.8-15.3	21.5	20.2-22.9
2013	0.394	0.333-0.466	1.39	1.23-1.58	5.10	4.75-5.47	14.0	13.3-14.8	21.4	20.1-22.8
2014	0.340	0.280-0.412	1.48	1.30-1.68	4.79	4.43-5.17	14.8	14.0-15.6	21.0	19.6-22.4
2015	0.330	0.265-0.411	1.59	1.38-1.82	5.11	4.71-5.55	16.1	15.1-17.1	23.2	21.6-25.0
2016	0.339	0.262-0.439	1.61	1.37-1.89	5.73	5.22-6.29	14.3	13.2-15.4	23.6	21.7-25.8
2017	0.398	0.304-0.521	1.47	1.21-1.78	4.91	4.37-5.52	13.5	12.3-14.8	23.2	20.9-25.7
2018	0.309	0.221-0.433	1.56	1.28-1.91	4.83	4.25-5.49	13.2	11.9-14.6	21.5	19.1-24.2

Mendonça SC, *et al. BMJ Open* 2021; 10:e042518. doi: 10.1136/bmjopen-2020-042518

Men – Observed rates										
	40-54		55-64		65-74		75-84		85+	
Year	Rate	95% CI	Rate	95% CI	Rate	95% CI	Rate	95% CI	Rate	95% CI
2004	0.824	0.738-0.920	3.13	2.91-3.38	8.52	8.06-9.02	17.0	16.1-17.9	22.2	20.2-24.5
2005	0.870	0.783-0.967	3.01	2.79-3.25	8.44	7.98-8.93	16.8	15.9-17.7	20.1	18.2-22.2
2006	0.750	0.669-0.839	3.22	2.99-3.46	8.40	7.93-8.88	17.9	17.0-18.9	23.1	21.1-25.3
2007	0.773	0.692-0.863	3.08	2.86-3.31	8.58	8.12-9.07	16.9	16.0-17.8	24.0	22.0-26.1
2008	0.770	0.689-0.859	3.21	2.99-3.45	8.41	7.95-8.89	16.7	15.9-17.6	22.1	20.2-24.1
2009	0.806	0.724-0.898	3.15	2.92-3.39	8.31	7.86-8.79	16.7	15.8-17.6	24.3	22.4-26.4
2010	0.725	0.647-0.813	2.99	2.77-3.23	8.28	7.83-8.76	17.5	16.6-18.4	24.8	22.9-27.0
2011	0.828	0.742-0.923	3.14	2.91-3.39	8.28	7.82-8.76	18.4	17.4-19.4	25.5	23.5-27.7
2012	0.834	0.748-0.931	3.36	3.12-3.63	9.15	8.67-9.66	19.1	18.2-20.1	25.4	23.4-27.6
2013	0.839	0.749-0.940	3.31	3.06-3.59	8.55	8.08-9.05	19.4	18.4-20.4	26.6	24.5-28.8
2014	0.901	0.802-1.013	3.28	3.01-3.58	9.15	8.63-9.70	19.2	18.2-20.3	27.6	25.4-30.1
2015	1.015	0.897-1.149	3.67	3.35-4.01	8.79	8.22-9.39	19.9	18.7-21.1	28.2	25.7-30.9
2016	0.927	0.795-1.082	3.22	2.87-3.60	8.79	8.12-9.52	19.4	18.0-21.0	27.3	24.4-30.5
2017	0.832	0.692-1.000	3.65	3.24-4.12	9.52	8.71-10.4	20.8	19.1-22.7	27.8	24.4-31.6
2018	0.952	0.788-1.150	3.17	2.76-3.65	9.32	8.46-10.3	19.1	17.3-21.0	27.6	24.0-31.8

Both genders – Modelled rates[(1)]										
	40-54		55-64		65-74		75-84		85+	
Year	Rate	95% CI	Rate	95% CI	Rate	95% CI	Rate	95% CI	Rate	95% CI
2004	0.594	0.540-0.652	2.38	2.24-2.54	6.76	6.47-7.06	15.1	14.5-15.6	20.8	19.7-22.0
2005	0.604	0.550-0.661	2.38	2.24-2.52	6.61	6.32-6.91	14.5	14.0-15.0	19.6	18.6-20.7
2006	0.588	0.536-0.644	2.39	2.25-2.54	6.85	6.56-7.15	15.3	14.7-15.8	20.8	19.7-21.9
2007	0.558	0.507-0.612	2.28	2.14-2.42	6.57	6.28-6.86	14.8	14.3-15.4	20.6	19.6-21.7
2008	0.586	0.534-0.641	2.35	2.22-2.50	6.69	6.40-6.98	15.0	14.4-15.5	20.7	19.7-21.7
2009	0.550	0.500-0.604	2.25	2.12-2.40	6.53	6.25-6.82	15.0	14.4-15.5	21.3	20.3-22.4
2010	0.528	0.479-0.581	2.20	2.07-2.35	6.47	6.19-6.76	14.8	14.2-15.3	20.5	19.5-21.6
2011	0.565	0.513-0.620	2.34	2.19-2.49	6.82	6.52-7.12	15.5	14.9-16.1	21.6	20.5-22.7
2012	0.595	0.541-0.653	2.45	2.30-2.61	7.15	6.85-7.45	16.4	15.8-17.0	23.2	22.1-24.3
2013	0.570	0.515-0.628	2.38	2.22-2.54	6.99	6.69-7.30	16.1	15.5-16.7	22.7	21.6-23.9
2014	0.581	0.523-0.645	2.42	2.25-2.59	7.09	6.77-7.42	16.3	15.7-17.0	23.1	21.9-24.4
2015	0.621	0.553-0.695	2.55	2.35-2.75	7.42	7.06-7.80	17.1	16.4-17.9	24.7	23.3-26.1
2016	0.589	0.511-0.676	2.45	2.23-2.69	7.24	6.81-7.69	16.8	15.9-17.7	24.0	22.3-25.7
2017	0.595	0.506-0.694	2.48	2.23-2.76	7.30	6.81-7.83	16.8	15.8-17.9	23.8	21.9-25.8
2018	0.585	0.489-0.694	2.41	2.14-2.70	7.02	6.49-7.59	16.1	15.0-17.2	22.6	20.5-24.7

Women – Modelled rates[(1)]										
	40-54		55-64		65-74		75-84		85+	
Year	Rate	95% CI	Rate	95% CI	Rate	95% CI	Rate	95% CI	Rate	95% CI
2004	0.350	0.292-0.416	1.63	1.46-1.81	5.30	4.95-5.67	13.4	12.8-14.1	20.5	19.2-21.8
2005	0.350	0.293-0.415	1.60	1.44-1.78	5.14	4.79-5.50	12.8	12.2-13.5	19.3	18.0-20.5
2006	0.378	0.319-0.445	1.71	1.54-1.88	5.40	5.04-5.77	13.3	12.7-14.0	19.7	18.5-21.0
2007	0.322	0.268-0.383	1.51	1.35-1.68	4.97	4.63-5.33	12.8	12.1-13.4	19.7	18.5-21.0
2008	0.346	0.291-0.409	1.61	1.45-1.78	5.25	4.91-5.62	13.3	12.7-14.0	20.3	19.1-21.6
2009	0.296	0.245-0.355	1.44	1.29-1.60	4.92	4.58-5.27	13.1	12.4-13.8	20.9	19.7-22.2
2010	0.318	0.265-0.380	1.48	1.33-1.65	4.87	4.54-5.22	12.4	11.8-13.1	19.1	17.9-20.4
2011	0.340	0.284-0.404	1.59	1.42-1.77	5.22	4.87-5.59	13.3	12.6-14.0	20.4	19.1-21.7
2012	0.330	0.274-0.394	1.59	1.42-1.78	5.38	5.03-5.75	14.2	13.4-14.9	22.4	21.1-23.8
2013	0.325	0.268-0.391	1.55	1.38-1.74	5.23	4.87-5.60	13.7	12.9-14.5	21.5	20.2-23.0
2014	0.313	0.253-0.383	1.52	1.33-1.72	5.17	4.79-5.57	13.7	12.9-14.6	21.9	20.5-23.5
2015	0.318	0.251-0.397	1.58	1.37-1.81	5.51	5.08-5.97	14.9	14.0-15.9	24.3	22.6-26.1
2016	0.339	0.258-0.439	1.64	1.39-1.92	5.55	5.04-6.10	14.7	13.6-15.8	23.3	21.3-25.4
2017	0.308	0.221-0.418	1.51	1.24-1.83	5.18	4.60-5.80	13.8	12.6-15.2	22.2	19.9-24.6
2018	0.300	0.207-0.422	1.46	1.17-1.80	5.00	4.38-5.67	13.3	11.9-14.7	21.1	18.7-23.8
Men – Modelled rates[(1)]										
	40-54		55-64		65-74		75-84		85+	
Year	Rate	95% CI	Rate	95% CI	Rate	95% CI	Rate	95% CI	Rate	95% CI
2004	0.829	0.740-0.925	3.14	2.91-3.39	8.37	7.90-8.86	17.3	16.4-18.3	21.6	19.5-23.8
2005	0.847	0.759-0.943	3.15	2.92-3.40	8.24	7.77-8.72	16.8	15.9-17.7	20.5	18.5-22.6
2006	0.792	0.707-0.884	3.08	2.86-3.32	8.45	7.98-8.94	18.0	17.1-19.0	23.1	21.0-25.2
2007	0.787	0.703-0.878	3.06	2.83-3.29	8.33	7.87-8.82	17.7	16.8-18.6	22.6	20.6-24.7
2008	0.816	0.731-0.908	3.10	2.87-3.33	8.28	7.82-8.76	17.2	16.3-18.1	21.5	19.6-23.5
2009	0.797	0.713-0.888	3.07	2.84-3.30	8.31	7.85-8.79	17.5	16.6-18.5	22.2	20.3-24.3
2010	0.732	0.652-0.821	2.93	2.71-3.17	8.23	7.77-8.71	18.0	17.1-19.0	23.7	21.8-25.8
2011	0.782	0.696-0.874	3.09	2.85-3.34	8.58	8.11-9.08	18.6	17.6-19.6	24.1	22.2-26.3
2012	0.850	0.760-0.947	3.31	3.06-3.58	9.10	8.61-9.60	19.4	18.4-20.4	25.0	22.9-27.1
2013	0.806	0.715-0.905	3.20	2.94-3.47	8.94	8.44-9.45	19.4	18.4-20.5	25.4	23.3-27.6
2014	0.843	0.744-0.952	3.32	3.04-3.62	9.21	8.68-9.76	19.9	18.8-21.0	25.8	23.6-28.1
2015	0.910	0.795-1.037	3.52	3.20-3.86	9.53	8.93-10.16	20.1	18.9-21.4	25.5	23.1-28.2
2016	0.829	0.699-0.976	3.28	2.92-3.67	9.11	8.41-9.84	19.7	18.3-21.2	25.6	22.7-28.7
2017	0.876	0.725-1.048	3.46	3.04-3.92	9.65	8.82-10.53	21.0	19.2-22.8	27.4	24.0-31.2
2018	0.854	0.692-1.043	3.35	2.91-3.84	9.25	8.37-10.20	19.9	18.0-21.9	25.7	22.1-29.7

(1) Modelled rates take into account the effect of changes in the age-gender case mix over time.

Mendonça SC, *et al. BMJ Open* 2021; 10:e042518. doi: 10.1136/bmjopen-2020-042518

Appendix 6: Modelled incidence of recorded AF over time by patient deprivation (most vs least deprived quintiles of the Index of Multiple Deprivation (IMD). The effect of deprivation was shown to be statistically significant (p=0.002, see also appendix 7).

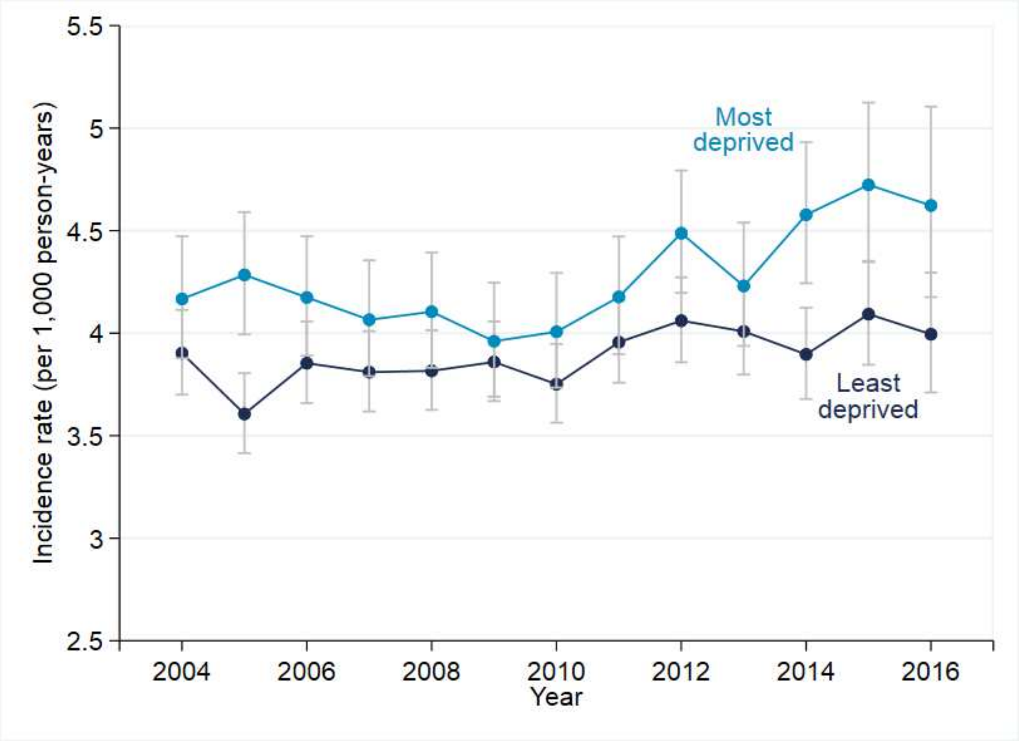

Appendix 7: Model results for the association between the Index of Multiple Deprivation (IMD) and incidence rates. Relative differences between deprivation categories are shown as incidence rate ratios (IRR).

	IRR (95% CI)	p
IMD (ref=1, least deprived)		0.002
2	1.06 (1.02-1.10)	
3	1.07 (1.01-1.14)	
4	1.10 (1.01-1.20)	
5, most deprived	1.17 (1.05-1.31)	
c.IMD*i.year (ref=2016)		0.182
2004	0.98 (0.95-1.01)	
2005	1.01 (0.97-1.04)	
2006	0.98 (0.95-1.02)	
2007	0.98 (0.95-1.01)	
2008	0.98 (0.95-1.02)	
2009	0.97 (0.94-1.00)	
2010	0.98 (0.95-1.01)	
2011	0.98 (0.95-1.01)	
2012	0.99 (0.96-1.02)	
2013	0.98 (0.95-1.01)	
2014	1.00 (0.97-1.04)	
2015	1.00 (0.97-1.04)	

Notes:

1) The model structure is as follows (as described in the methods section):
Year + agecat + gender + IMD + i.year*c.agecat + i.year*i.gender + i.year*c.IMD + c.agecat*i.gender + i.year*c.agecat*i.gender

2) This analysis uses a subsample of patients with complete IMD during the period 2004 to 2016. It includes a total of 74,514 AF incident cases and 17.8 million person-years of observation. Due to methodological differences between the IMD stratified denominator provided by CPRD and the way we have calculated the incidence rates, the rate for all patients in this subsample is slightly lower than our main analysis rate. Therefore we prefer to show relative differences (as incidence rate ratios, IRR) instead of absolute differences in the quintiles of IMD.

Mendonça SC, *et al. BMJ Open* 2021; 10:e042518. doi: 10.1136/bmjopen-2020-042518

Appendix 8: Observed incidence rates (per 1,000 person-years) for recorded AF during the period 2004-2018. This is an additional stratification by age (under and over 65), for comparison with other studies.

	Incidence rate	95% CI
40-64	1.21	1.19-1.23
65+	11.6	11.5-11.6
Women		
40-64	0.75	0.73-0.77
65+	10.4	10.3-10.5
Men		
40-64	1.66	1.63-1.69
65+	13.0	12.9-13.2

Mendonça SC, *et al. BMJ Open* 2021; 10:e042518. doi: 10.1136/bmjopen-2020-042518
