# Supplementary material for: Temporal trends in incidence of atrial fibrillation in primary care records: a population-based cohort study
Source: BMJ Open. 2020 Dec 23;10(12):e042518. doi: 10.1136/bmjopen-2020-042518 (PMC7759967; doi:10.1136/bmjopen-2020-042518)
Supplement: Author's manuscript [file bmjopen-2020-042518.draft_revisions.pdf]

BMJ Open

BMJ Open is committed to open peer review. As part of this commitment we make the peer review history of every article we publish publicly available.

When an article is published we post the peer reviewers' comments and the authors' responses online. We also post the versions of the paper that were used during peer review. These are the versions that the peer review comments apply to.

The versions of the paper that follow are the versions that were submitted during the peer review process. They are not the versions of record or the final published versions. They should not be cited or distributed as the published version of this manuscript.

BMJ Open is an open access journal and the full, final, typeset and author-corrected version of record of the manuscript is available on our site with no access controls, subscription charges or pay-per-view fees (http://bmjopen.bmj.com).

If you have any questions on BMJ Open's open peer review process please email info.bmjopen@bmj.com

BMJ Open

Temporal trends in incidence of atrial fibrillation in primary care records

Journal:	*BMJ Open*
Manuscript ID	bmjopen-2020-042518
Article Type:	Original research
Date Submitted by the Author:	07-Jul-2020

Complete List of Authors:	Mendonca, Silvia; University of Cambridge, Primary Care Unit, Department of Public Health & Primary Care<br>Saunders, Catherine; University of Cambridge, Primary Care Unit, Department of Public Health & Primary Care<br>Lund, Jenny; University of Cambridge, Primary Care Unit, Department of Public Health & Primary Care<br>Mant, Jonathan; University of Cambridge, Primary Care Unit, Department of Public Health & Primary Care<br>Edwards, Duncan; University of Cambridge, Primary Care Unit, Department of Public Health & Primary Care

Keywords:	PRIMARY CARE, EPIDEMIOLOGY, Health policy < HEALTH SERVICES ADMINISTRATION & MANAGEMENT

SCHOLARONE™
Manuscripts

I, the Submitting Author has the right to grant and does grant on behalf of all authors of the Work (as defined in the below author licence), an exclusive licence and/or a non-exclusive licence for contributions from authors who are: i) UK Crown employees; ii) where BMJ has agreed a CC-BY licence shall apply, and/or iii) in accordance with the terms applicable for US Federal Government officers or employees acting as part of their official duties; on a worldwide, perpetual, irrevocable, royalty-free basis to BMJ Publishing Group Ltd ("BMJ") its licensees and where the relevant Journal is co-owned by BMJ to the co-owners of the Journal, to publish the Work in this journal and any other BMJ products and to exploit all rights, as set out in our licence.

The Submitting Author accepts and understands that any supply made under these terms is made by BMJ to the Submitting Author unless you are acting as an employee on behalf of your employer or a postgraduate student of an affiliated institution which is paying any applicable article publishing charge ("APC") for Open Access articles. Where the Submitting Author wishes to make the Work available on an Open Access basis (and intends to pay the relevant APC), the terms of reuse of such Open Access shall be governed by a Creative Commons licence – details of these licences and which Creative Commons licence will apply to this Work are set out in our licence referred to above.

Other than as permitted in any relevant BMJ Author's Self Archiving Policies, I confirm this Work has not been accepted for publication elsewhere, is not being considered for publication elsewhere and does not duplicate material already published. I confirm all authors consent to publication of this Work and authorise the granting of this licence.

For peer review only

Title: Temporal trends in incidence of atrial fibrillation in primary care records

Co-authors: Sílvia C Mendonça, Catherine L Saunders, Jenny Lund, Jonathan Mant, Duncan Edwards

Primary Care Unit, Department of Public Health and Primary Care, University of Cambridge, Institute of Public Health, Forvie Site, Robinson Way, Cambridge, CB2 0SR

Correspondence: Silvia Mendonca

Institute of Public Health, Forvie Site, Robinson Way, Cambridge, CB2 0SR

sm2061@medschl.cam.ac.uk

For peer review only

ABSTRACT

Objectives Atrial fibrillation (AF) is a heart condition associated with a fivefold increased risk of stroke. The condition is easy to detect in primary care and treatment can greatly reduce the risk of stroke. In recent years, a number of policy initiatives have tried to improve diagnosis and treatment of AF, including local NHS schemes and the Quality and Outcomes Framework. We aimed to examine trends in the incidence of recorded AF in primary care records from English practices between 2004 and 2018.

Design Longitudinal cohort study.

Setting English primary care electronic health records linked to Index of Multiple Deprivation data.

Participants Cohort of 3.5 million patients over 40 years old registered in general practices in England, contributing 22 million person-years of observation between 2004 and 2018.

Primary and secondary outcome measures Incident AF was identified through newly recorded AF codes in the patients' records. Yearly incidence rates were stratified by gender, age group and a measure of deprivation.

Results Incidence rates were stable before 2010 and then rose and peaked in 2015 at 5.1 (95% CI 4.94-5.20) cases per 1,000 person-years. Incidence was higher in males (4.95 [95% CI 4.91-4.99] cases per 1,000 person-years vs 4.12 [95% CI 4.08-4.16] in females) and rises markedly with age (0.58 [95% CI 0.56-0.59] cases per 1,000 person-years in 40-54 years old vs 21.7 [95% CI 21.4-22.0] cases in over 85s). The increase in incidence over time was observed mainly in people over the age of 75, particularly men. There was no evidence that incidence was associated with deprivation.

Conclusions Changes in clinical practice and policy initiatives since 2004 have been associated with increased rates of diagnosis of AF up until 2015, but rates declined from 2015 to 2018.

STRENGTHS AND LIMITATIONS OF THIS STUDY

- Our population based sample covered 3.5 million patients at risk over a period of 15 years.
- The data from the Clinical Practice Research Datalink (CPRD) used in this study have been shown to be representative of the UK population and to be of high quality for the recording of diagnoses, particularly for chronic conditions.
- Atrial fibrillation has been part of the Quality and Outcomes Framework since 2007 and is likely to be well recorded in primary care records.
- A limitation is that these reported incidence rates exclude patients with undiagnosed AF, or that don't have their diagnosis recorded in their primary care records.

INTRODUCTION

Atrial fibrillation is a cardiac arrhythmia that is associated with a five-fold increase in risk of stroke[1]. The risk of stroke associated with AF can be greatly reduced by use of anticoagulants. AF is associated with 1 in 5 stroke cases[2] and is considered to be underdiagnosed[3] and undertreated[4]. Consequently, AF has been the target of a number of policy initiatives in recent years aimed at both treatment and diagnosis. In the UK, it has been part of the Quality and Outcomes Framework (QOF) since 2006[5], the pay for performance scheme that incentivizes the recording and appropriate management of chronic conditions. Since 2008 there have been several local NHS schemes to incentivize detection and management of AF including the GRASP-AF electronic tool[6] and opportunistic screening by manual pulse checks[5]. It is the subject of a national programme co-ordinated by the Academic Health Sciences Network[2]. The need for better detection is highlighted in the NHS Long Term Plan (2019)[7]. There is evidence from both UK and international studies of a rise in the age-specific incidence of AF[8,9], including two using UK electronic primary care records[10,11]. However, these have not used data beyond 2011. Given that policy initiatives continue to focus on AF, our aim was to describe temporal trends in recorded AF in a nationally representative sample of primary care records up until 2018.

METHODS

Data source

We used data from the Clinical Practice Research Datalink (CPRD) GOLD, a database of electronic primary care records in the UK, based on patients attending GP practices which use the Vision computer system. CPRD has been shown to be nationally representative of the UK population[12] and data quality is monitored at the practice and patient level. In 2013 it contained the medical records of over 11 million patients, with 4.4 active (currently registered) patients corresponding to about 7% of the UK population[12]. During the study period (2004 to 2018) the number of UK practices contributing data to CPRD varied from 680 to 367 (Appendix 1) due to a reduction of practices using the Vision computer system. The data collected from CPRD include all clinical codes for medical diagnoses entered at consultations, referrals, tests and all prescriptions issued at the practice.

For peer review only - http://bmjopen.bmj.com/site/about/guidelines.xhtml

Study cohort and disease definition

The study population included patients from English practices aged over 40 years old between the 1st January 2004 and 31st December 2018. Recorded AF was identified using a list of 8 Read codes (Appendix 2). This list was developed as part of a project on the epidemiology of multimorbidity[13,14] and was based originally on the QOF business rules. We used the October 2019 data release of CPRD GOLD.

Data analysis

AF incidence was estimated as the number of new AF cases during each year, divided by the eligible follow up (person-time at risk). Eligible follow up for all patients (with and without AF) was calculated using the CPRD denominator files, which contain basic information about each patient and practice. Start of follow up was calculated as the latest of: one year after the patient first registered at the practice, the practice up-to-standard date (an internal measure of CPRD data quality, based on consistency of data provided to CPRD by the practice[12]) and 1st of January 2004 (study start). The end of follow-up was the earliest of: the last date the patient was registered at the practice, date of death, the last date the practice contributed data to CPRD, or the 31st of December 2018. We restricted follow-up to start one year after registration at the practice to avoid capturing historical diagnosis.

People with a new AF medical code recorded for the first time during the eligible follow up period were identified as incident cases and counted towards the numerator. Those people with any AF code identified before the start of eligible follow up were considered prevalent cases and were excluded. Cases registered after the end of eligible follow up were also excluded from the numerator, except for a few cases that occurred within 3 months of death or transfer out date. We decided to include these as they probably relate to events that occurred within eligible follow up but that were registered later. AF cases that occurred within or after eligible follow up contribute time at risk to the denominator until their date of diagnosis or end of follow up, whichever is first.

Incidence rates are calculated as the number of new cases of AF in our eligible population and study period (numerator) divided by the total person time at risk (denominator). The denominator was the sum of the person-time at risk for all eligible patients (cases and non-cases).

The patients' sex and year of birth were available from CPRD. We assumed the patients' date of birth to be the mid-year (i.e. the 30th of June) of year of birth. Patients' age were grouped into 5 categories: 40 to 54, 55 to 64, 65 to 74, 75 to 84 and ≥85 years old.

Stratified incidence rates by calendar year and socio-demographic characteristics were calculated through the use of a Lexis expansion[15]. Each patient time at risk was split into multiple time periods according to their age group and calendar years. Time at risk is then added up within each strata, a combination of calendar year by age group by gender (eg, females aged 65 to 74 in 2016). The incidence rate corresponds to the number of AF cases divided by the total person-time at risk in that strata.

In order to take into account possible changes over time in the age composition of the sample we have additionally estimated modelled incidence rates. We used a poisson regression approach using the number of events in each strata as the main outcome and including the logarithm of follow up time as an offset. The model for all patients included the main effect variables year, age group and sex and their two-way and three-way interactions. Year and sex were always treated as categorical variables but age group was treated as a continuous variable for the interaction terms

For peer review only - http://bmjopen.bmj.com/site/about/guidelines.xhtml

only in order to maximize power. Each model was used to predict the incidence rate for each year adjusting for the case-mix characteristics of the whole sample (known as marginal standardization)[16].

Incidence by deprivation was calculated using a CPRD patient denominator file stratified by the patient level 2010 English Index of Multiple Deprivation (IMD) quintiles and the linked patient level IMD quintiles for the patients with newly registered AF. For this data linkage, data are only available until the end of 2016. To model the effect of deprivation we used the same model as the main analysis but adding in a categorical main effect for deprivation quintile and an interaction between year and deprivation quintile (treated as a linear term).

Data manipulation and analyses were performed in Stata (StataCorp, version 15.1). We report 95% confidence intervals calculated as exact Poisson confidence intervals.

Patient and Public Involvement

Patients and the public were not involved in this study.

RESULTS

In a study population of nearly 3.5 million patients contributing a total of 22 million person-years of observation, there were 99,836 newly registered AF cases (see patient selection flowchart in Appendix 3) with a mean age at diagnosis of 74.6 years (see Table 1). Over 55% of new diagnoses were in people over the age of 75. The overall incidence is 4.52 cases per 1,000 patient years in people aged over 40 years old, and 11.6 per 1,000 patient years in people aged 65 years and older. The incidence rises by age, up to 21.7 per 1,000 in people aged 85 or over, and is higher in men than women, 4.95 versus 4.12 per 1,000 person years (Table 2).

Incidence of AF was relatively stable between 2004 and 2010, between 4.2 and 4.5 cases per 1,000 person-years, then increased up until 2015, when incidence peaked at 5.1 cases per 1,000 person-years, before falling back to 4.6 cases per 1,000 person years by 2018 (see figure 1 and appendix 4). This post-2015 decline occurred primarily in women. The age-specific trends show that the increase between 2010 and 2015 occurred largely in people aged 75 and over, and more so in men (figure 2 and appendix 5).

Incidence of AF was higher in the most deprived quintile of IMD than in the least deprived quintile (Appendix 6 and 7). There was no evidence of differences in temporal trends in AF by levels of socio economic deprivation.

DISCUSSION

SUMMARY

We observed stable AF incidence from 2004 to 2010, increased incidence from 2010 to 2015, and then falling incidence 2016 to 2018. The rise in incidence was primarily in people aged 75 and over and most marked in men. The decline from 2016 to 2018 reflected falling incidence in women rather than men.

STRENGTHS AND LIMITATIONS

For peer review only - http://bmjopen.bmj.com/site/about/guidelines.xhtml

A strength of this study was the use of a large sample that is representative of the English population, permitting a detailed description of current practice. It is important to note that the focus of this study was *recorded* (or clinically detected) atrial fibrillation, and thus patients with undiagnosed, or who are diagnosed but not recorded will not be included. However, diagnosis and inclusion in population registers is essential to ensure adequate care and stroke prevention, and the focus of this study was to describe the incidence of clinically detected AF. Our modelled incidence rates remove the effect that possible changes in the population case-mix over time could have on observed rates. However, changes in the number of practices using the Vision computer system in recent years could be contributing to the levelling off in incidence rates observed after 2015[17].

COMPARISON WITH EXISTING LITERATURE

Incidence rates for AF can vary considerably between different studies, most likely due to different source populations, case definitions and study periods. The rates reported in this study are similar to those described in other European cohorts from Iceland[18] and the Netherlands[19] but lower than one from Germany[20]. In general, incidence rates reported from American cohorts[21-23] are higher than those from Europe, including our own study.

In the UK population, we found two studies that looked at incidence of AF over time[10,11]. Scowcroft et al. calculated incidence rates between 2000 and 2012 while Lane et al. reports incidence rates between 1998 and 2010. Both studies use data from CPRD and also report increases in incidence (Scowcroft from 2009 onwards and Lane between the periods 1998-2001 and 2002-2006.) We found higher incidence rates than those reported by Lane and Scowcroft. Whereas we restricted our analysis to people aged 40 years and over, they both reported incidence in people aged 18 years and over. However, even when looking at comparable age groups, our rates were higher. For example, Lane et al reported an incidence rate of 3.26 per 1,000 people aged 65-74 between 2007 and 2010, whereas the observed incidence rate in our study in this age group was 6.84 per 1,000. This is likely to reflect analytical differences in how the denominator population was defined. In our study, we derived both the numerator and denominator by first defining the eligible follow up period for all patients and then identifying which AF cases fell in this eligibility period. In the other two UK studies these figures appear to have been derived separately, with stricter criteria applied to the cases (eg. Scowcroft et al. imposes a minimum of one year both pre and post diagnosis) than to the denominator, resulting in lower rates, though the observed temporal trends will not have been affected. We believe our estimates are truer reflections of the incidence of AF. A recent meta-analysis of AF screening studies in a general population estimated a detection rate of 1.44% in patients aged 65 and over[24]. This is slightly higher than our estimate of 1.16 cases per 100 person years in the comparable age group (see Appendix 8), which is what would be expected given that these were screening studies, whereas ours would have identified only those picked up from active case finding[3]. Further corroborative evidence that our incidence rates are more likely to be correct comes from the Framingham study, which reported incidence rates of 13.4 per 1,000 in men over the age of 50, and 8.6 per 1,000 in women. The higher rates in Framingham, despite the slightly younger age group, reflect the multiple sources of ascertainment that were used to identify AF. Detection rates from routine practice will be lower than those.

Ours is the first study to demonstrate a decline in the incidence of recorded AF between 2015-2018 in the UK, after taking account of changes over time in age composition of the population. Given that epidemiological studies such as Framingham have reported increases rather than decreases in AF incidence [9], this is likely to reflect a change in ascertainment in recent years.

For peer review only - http://bmjopen.bmj.com/site/about/guidelines.xhtml

IMPLICATIONS FOR RESEARCH, POLICY AND PRACTICE

Our results suggest that initiatives in policy and clinical practice since 2004 have been associated with increased rates of diagnosis of AF (Textbox 1). Although the increase in incidence seen in over 85 year olds may be explained in part by the increase mean age of this group over time, it is also plausible that initiatives to encourage pulse palpation in over 65s[5], or improved perceived tolerability of newer anticoagulants[25] have influenced clinicians to increase efforts to diagnose older patients. It is also notable that increases in AF diagnosis do not appear to have come at the expense of the widening social inequality that has been of particular concern in conditions such as cancer[26]. The small decline in AF incidence since 2015 may reflect reducing policy emphasis or a decline in the primary care workforce available to detect AF[27]. The decline in incidence may also reflect that "catch up" diagnoses were made in the years prior to 2015, boosting apparent incidence during that period. It is important to continue to monitor trends in AF incidence to assess impact of new initiatives. Our finding that incidence of AF in the under 65 age group remained low and did not change during the study period suggests first that there has been no substantive shift in the clinical presentation of this group to primary care that has led to changes in AF incidence in this population. Policy initiatives such as incentives for opportunistic screening have not focused on younger people with AF; our study would tend to support this approach as the numbers of people identified would be likely to be very low.

Our research has also highlighted the methodological point that researchers should ensure they use the correct denominators for studies of incidence in primary care database analyses as small changes in approaches can lead to substantial errors in published rates, with potentially large implications for policy decisions based on these estimates. We would recommend that policymakers base future modelling decisions and analyses on the rates presented in this, rather than previous studies.

CONCLUSION

The incidence of diagnosed AF increased in the UK between 2004-2015, with the greatest increase among patients aged over 85, but with a small decline between 2015-18. Trials of AF screening that are powered to detect impact on stroke rates will provide evidence on the extent to which efforts should be made to reverse this decline.[28]

Acknowledgements This study is based in part on data from the Clinical Practice Research Datalink obtained under licence from the UK Medicines and Healthcare products Regulatory Agency. The data is provided by patients and collected by the NHS as part of their care and support. The interpretation and conclusions contained in this study are those of the author/s alone. JM is an NIHR Senior Investigator.

Contributors DE and JM had the original idea for the study. DE, SM, CS, JL and JM designed the study. SM undertook data extraction and performed the analysis. SM and DE wrote the first draft of the paper, which was revised in collaboration with all authors. DE acts as guarantor.

Funding This report is independent research funded by the National Institute for Health Research (NIHR School for Primary Care Research, reference FR11/290). The views expressed in this

For peer review only - http://bmjopen.bmj.com/site/about/guidelines.xhtml

publication are those of the authors and not necessarily those of the NHS, the National Institute for Health Research or the Department of Health.

Competing interests All authors report grants from the NIHR School of Primary Care Research, during the conduct of the study. JL reports grants from the Wellcome Trust and the National Institute for Health Research, outside the submitted work. JM is on an advisory board for BMS/Pfizer on an AF screening trial, and is chief investigator of an NIHR funded programme grant on screening for atrial fibrillation. DE is a co-investigator on this same NIHR programme.

Ethics approval This study was approved by the Independent Scientific Advisory Board for Medicines and Healthcare products Regulatory Agency research (protocol reference number 17_079R). No further ethics approval is required.

Data sharing statement The study uses data from the Clinical Practice Research Datalink (CPRD). CPRD does not allow the sharing of patient-level data. The data specification for the CPRD dataset is available at: https://www.cprd.com/sites/default/files/CPRD%20GOLD%20Full%20Data%20Specification%20v2.0_0.pdf Additional information on the IMD patient level linkage data is available at: https://cprd.com/sites/default/files/Documentation_SmallAreaData_Patient_set18_v2.7.pdf. The code list used in this study is part of a set of lists made available at www.phpc.cam.ac.uk/pcu/cprd_cam/codelists/.

For peer review only - http://bmjopen.bmj.com/site/about/guidelines.xhtml

Table 1: Characteristics of patients with newly diagnosed AF in the period 2004 to 2018.

	N (%)
All patients	99836
Gender	
Men	52823 (52.9)
Women	47013 (47.1)
Age, mean (SD)	74.6 (11.2)
Age group	
40 - 54	5597 (5.6)
55 - 64	12598 (12.6)
65 - 74	26308 (26.4)
75 - 84	36215 (36.3)
85+	19118 (19.1)

Table 2: Observed incidence rates (per 1,000 person-years) for recorded AF during the period 2004 to 2018– stratified by age and gender.

	Incidence rate	95% CI
All patients	4.52	4.49-4.55
Gender		
Female	4.12	4.08-4.16
Male	4.95	4.91-4.99
Age group		
40 - 54	0.58	0.56-0.59
55 - 64	2.36	2.32-2.40
65 - 74	6.84	6.76-6.93
75 - 84	15.5	15.3-15.7
85+	21.7	21.4-22.0
Gender and age group		
Women		
40 - 54	0.32	0.30-0.34
55 - 64	1.52	1.48-1.57
65 - 74	5.24	5.14-5.34
75 - 84	13.7	13.5-13.9
85+	20.3	20.0-20.7
Men		
40 - 54	0.83	0.80-0.85
55 - 64	3.20	3.14-3.27
65 - 74	8.61	8.48-8.75
75 - 84	18.0	17.7-18.3
85+	24.8	24.2-25.3

For peer review only

Box 1: Key changes in policy and practice during the study period, 2004 to 2018.

Policy or Evidence Change	Year
Requirement for practice AF registers incorporated within QOF[29]	2007
First major NOAC RCT demonstrates effectiveness (RE-LY study)[30]	2009
Widespread introduction of local incentive schemes (LES) to diagnose and/or treat AF[31]	2011
Expansion of AF within QOF from three to four indicators[29]	2013
NICE guidance suggests a larger proportion of AF patients are eligible for anticoagulation[32]	2014

For peer review only

For peer review only - http://bmjopen.bmj.com/site/about/guidelines.xhtml

REFERENCES

1. Wolf PA, Abbott RD, Kannel WB. Atrial fibrillation as an independent risk factor for stroke: the Framingham Study. Stroke 1991;22:983-8.
2. The AHSN Network (2019) Atrial Fibrillation. . at https://www.ahsnnetwork.com/about-academic-health-science-networks/national-programmes-priorities/atrial-fibrillation.)
3. Fitzmaurice DA, Hobbs FD, Jowett S, et al. Screening versus routine practice in detection of atrial fibrillation in patients aged 65 or over: cluster randomised controlled trial. BMJ 2007;335:383.
4. Nieuwlaat R, Capucci A, Lip GY, et al. Antithrombotic treatment in real-life atrial fibrillation patients: a report from the Euro Heart Survey on Atrial Fibrillation. Eur Heart J 2006;27:3018-26.
5. Cole J, Torabi P, Dostal I, Homer K, Robson J. Opportunistic pulse checks in primary care to improve recognition of atrial fibrillation: a retrospective analysis of electronic patient records. Br J Gen Pract 2018;68:e388-e93.
6. Quality and Outcomes Framework - 2006-07. (Accessed 18.01.2020, at https://digital.nhs.uk/data-and-information/publications/statistical/quality-and-outcomes-framework-achievement-data/quality-and-outcomes-framework-2006-07.)
7. The NHS Long Term Plan (2019). (Accessed 15/02/2020, at https://www.longtermplan.nhs.uk/.)
8. Chugh SS, Havmoeller R, Narayanan K, et al. Worldwide epidemiology of atrial fibrillation: a Global Burden of Disease 2010 Study. Circulation 2014;129:837-47.
9. Schnabel RB, Yin X, Gona P, et al. 50 year trends in atrial fibrillation prevalence, incidence, risk factors, and mortality in the Framingham Heart Study: a cohort study. Lancet 2015;386:154-62.
10. Scowcroft AC, Cowie MR. Atrial fibrillation: improvement in identification and stroke preventive therapy - data from the UK Clinical Practice Research Datalink, 2000-2012. Int J Cardiol 2014;171:169-73.
11. Lane DA, Skjoth F, Lip GYH, Larsen TB, Kotecha D. Temporal Trends in Incidence, Prevalence, and Mortality of Atrial Fibrillation in Primary Care. J Am Heart Assoc 2017;6.
12. Herrett E, Gallagher AM, Bhaskaran K, et al. Data Resource Profile: Clinical Practice Research Datalink (CPRD). Int J Epidemiol 2015;44:827-36.
13. Cassell A, Edwards D, Harshfield A, et al. The epidemiology of multimorbidity in primary care: a retrospective cohort study. Br J Gen Pract 2018;68:e245-e51.
14. Payne RA, Mendonca SC, Elliott MN, et al. Development and validation of the Cambridge Multimorbidity Score. CMAJ 2020;192:E107-E14.
15. Clayton D, Hills M. Statistical models in epidemiology. Oxford ; New York: Oxford University Press; 1993.
16. Muller CJ, MacLehose RF. Estimating predicted probabilities from logistic regression: different methods correspond to different target populations. Int J Epidemiol 2014;43:962-70.
17. Kontopantelis E, Stevens RJ, Helms PJ, Edwards D, Doran T, Ashcroft DM. Spatial distribution of clinical computer systems in primary care in England in 2016 and implications for primary care electronic medical record databases: a cross-sectional population study. BMJ Open 2018;8:e020738.
18. Stefansdottir H, Aspelund T, Gudnason V, Arnar DO. Trends in the incidence and prevalence of atrial fibrillation in Iceland and future projections. Europace 2011;13:1110-7.
19. Heeringa J, van der Kuip DA, Hofman A, et al. Prevalence, incidence and lifetime risk of atrial fibrillation: the Rotterdam study. Eur Heart J 2006;27:949-53.
20. Wilke T, Groth A, Mueller S, et al. Incidence and prevalence of atrial fibrillation: an analysis based on 8.3 million patients. Europace 2013;15:486-93.
21. Krahn AD, Manfreda J, Tate RB, Mathewson FA, Cuddy TE. The natural history of atrial fibrillation: incidence, risk factors, and prognosis in the Manitoba Follow-Up Study. Am J Med 1995;98:476-84.

For peer review only - http://bmjopen.bmj.com/site/about/guidelines.xhtml

22. Psaty BM, Manolio TA, Kuller LH, et al. Incidence of and risk factors for atrial fibrillation in older adults. Circulation 1997;96:2455-61.
23. Benjamin EJ, Levy D, Vaziri SM, D'Agostino RB, Belanger AJ, Wolf PA. Independent risk factors for atrial fibrillation in a population-based cohort. The Framingham Heart Study. JAMA 1994;271:840-4.
24. Lowres N, Olivier J, Chao TF, et al. Estimated stroke risk, yield, and number needed to screen for atrial fibrillation detected through single time screening: a multicountry patient-level meta-analysis of 141,220 screened individuals. PLoS Med 2019;16:e1002903.
25. Wilke T, Bauer S, Mueller S, Kohlmann T, Bauersachs R. Patient Preferences for Oral Anticoagulation Therapy in Atrial Fibrillation: A Systematic Literature Review. Patient 2017;10:17-37.
26. Hirst Y, Stoffel S, Baio G, McGregor L, von Wagner C. Uptake of the English Bowel (Colorectal) Cancer Screening Programme: an update 5 years after the full roll-out. Eur J Cancer 2018;103:267-73.
27. General Practice Workforce. 2020. (Accessed 06/06/2020, at https://digital.nhs.uk/data-and-information/publications/statistical/general-and-personal-medical-services/final-31-december-2019.)
28. Lown M, Garrard J, Irving G, Edwards D, Hobbs FR, Mant J. Should we screen for atrial fibrillation? British Journal of General Practice 2017;67:296-7.
29. QOF database. (Accessed 21/02/2020, at https://www.gpcontract.co.uk/.)
30. Connolly SJ, Ezekowitz MD, Yusuf S, et al. Dabigatran versus warfarin in patients with atrial fibrillation. N Engl J Med 2009;361:1139-51.
31. Robson J, Dostal I, Mathur R, et al. Improving anticoagulation in atrial fibrillation: observational study in three primary care trusts. Br J Gen Pract 2014;64:e275-e81.
32. Senoo K, Lau YC, Lip GY. Updated NICE guideline: management of atrial fibrillation (2014). Expert Rev Cardiovasc Ther 2014;12:1037-40.

For peer review only

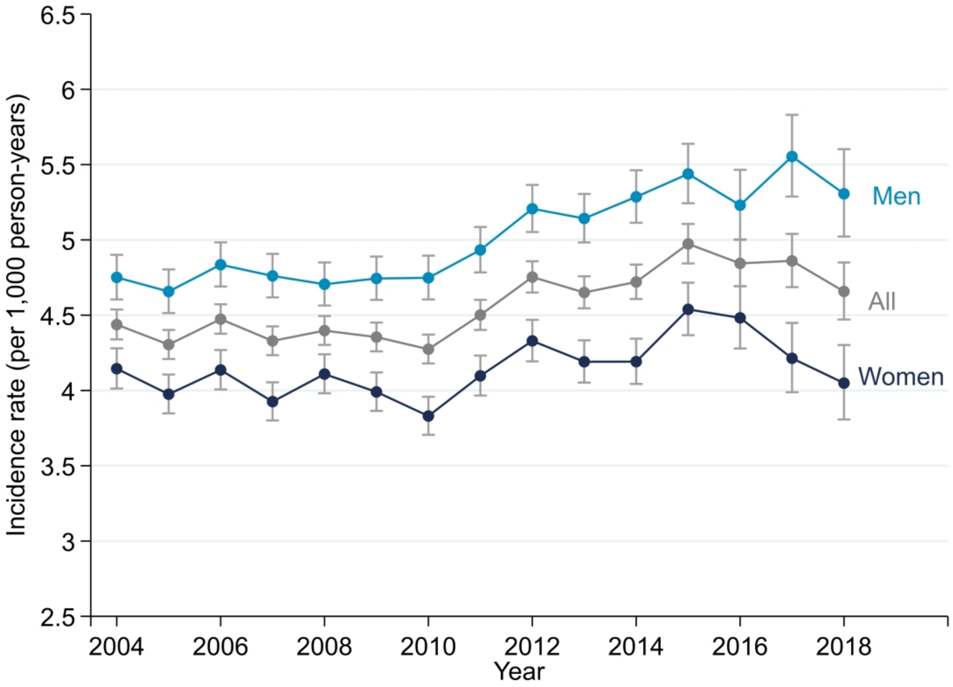

Figure 1: Modelled incidence of recorded AF over time.

1411x1026mm (72 x 72 DPI)

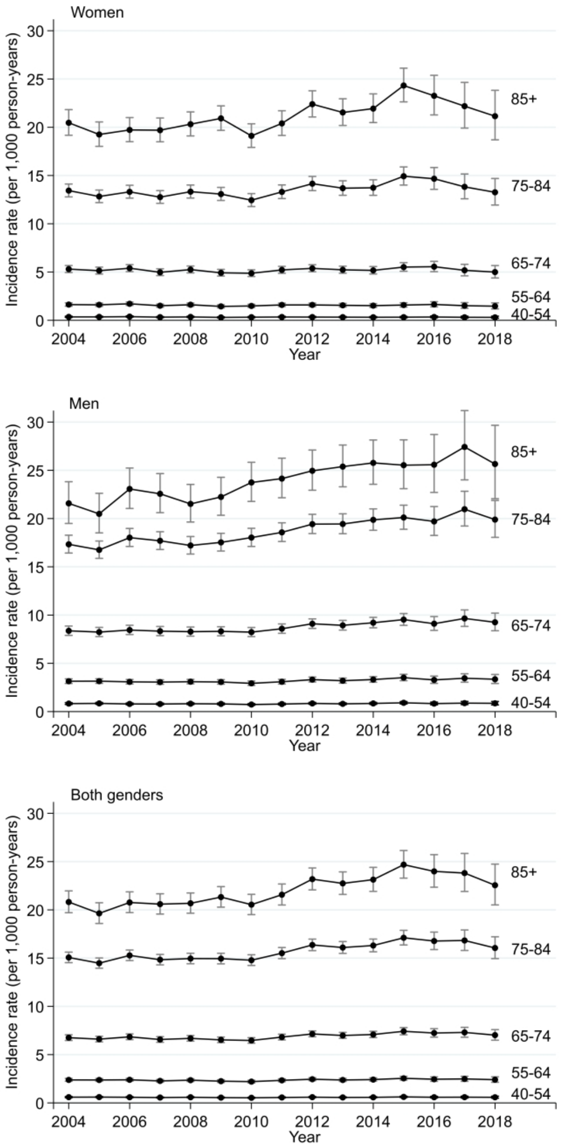

Figure 2: Modelled incidence of recorded AF over time stratified by age group and gender.

1411x2822mm (72 x 72 DPI)

Appendix 1: Number of practices contributing data to CPRD GOLD during study period. Figures are mid-year counts.

	2004	2006	2008	2010	2012	2014	2016	2018
UK practices	680	708	717	703	672	599	448	367
English practices	451	460	462	443	411	335	185	110
Scottish, Welsh and Northern Irish practices	229	248	255	260	261	264	263	257

Appendix 2: List of codes used to define Atrial Fibrillation. The presence of these codes in the patient record, in the clinical and referral tables, was considered as evidence of diagnosis.

Medcode	Readcode	Readterm
1664	G573000	Atrial fibrillation
2212	G573.00	Atrial fibrillation and flutter
1268	G573200	Paroxysmal atrial fibrillation
1757	G573100	Atrial flutter
23437	G573z00	Atrial fibrillation and flutter NOS
96076	G573500	Persistent atrial fibrillation
96277	G573400	Permanent atrial fibrillation
35127	G573300	Non-rheumatic atrial fibrillation

For peer review only

Appendix 3: Flowchart for cohort - incident cases of AF from English practices.

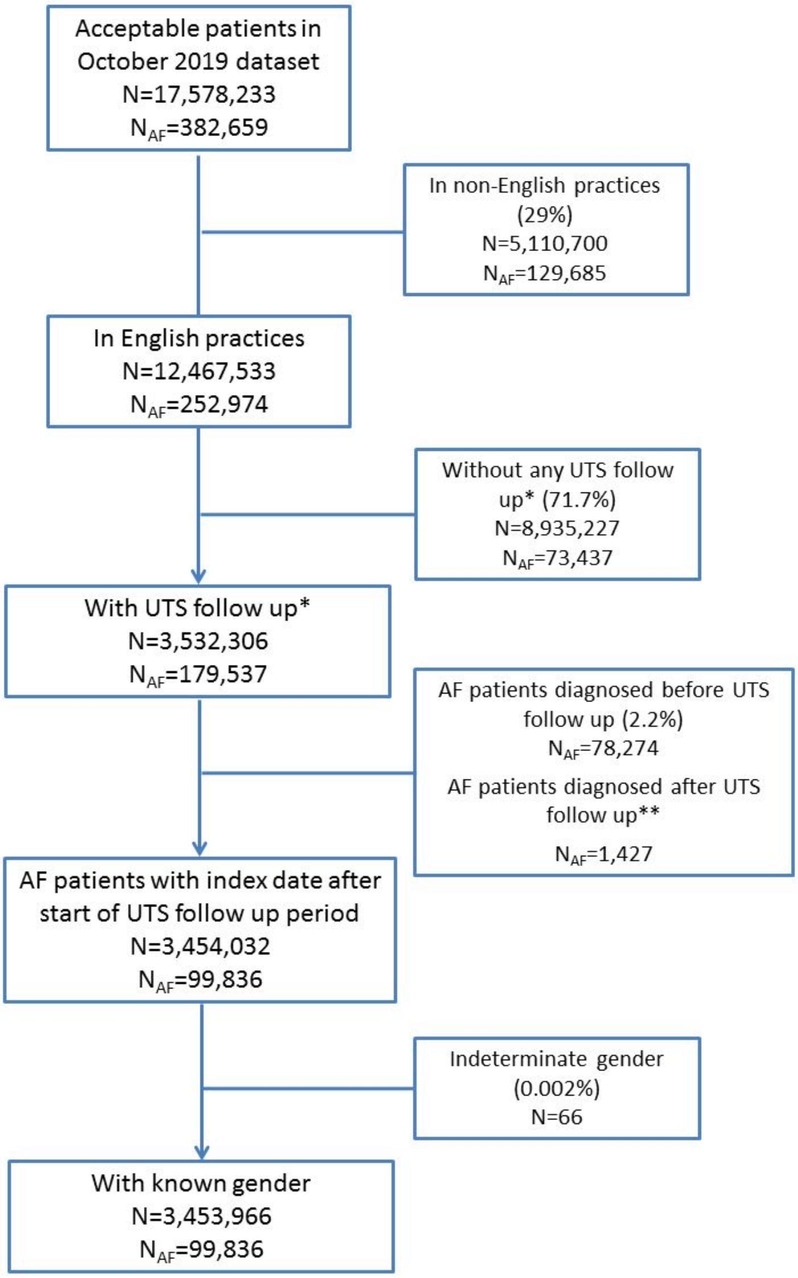

Notes: Each step shows the total number of patients in the denominator (N) and the sub-sample with AF in the numerator (N_{AF}). *"Up-to-standard" (UTS) follow up as defined in the methods section. ** N patients to be excluded from numerator but will contribute to denominator.

Appendix 4: Observed and modelled incidence rates (per 1,000 person-years) for recorded AF during the period 2004-2018– stratified by calendar year and gender.

Observed rates						
	Both genders		Women		Men	
Year	Rate	95% CI	Rate	95% CI	Rate	95% CI
2004	4.46	4.36-4.56	4.23	4.10-4.37	4.71	4.57-4.86
2005	4.31	4.21-4.40	4.03	3.90-4.16	4.61	4.46-4.75
2006	4.46	4.36-4.56	4.17	4.05-4.31	4.77	4.62-4.91
2007	4.31	4.22-4.41	3.95	3.83-4.08	4.69	4.55-4.84
2008	4.37	4.28-4.47	4.11	3.99-4.24	4.64	4.51-4.79
2009	4.32	4.23-4.42	3.98	3.85-4.10	4.69	4.55-4.84
2010	4.24	4.15-4.34	3.81	3.68-3.94	4.71	4.57-4.86
2011	4.49	4.39-4.59	4.07	3.94-4.21	4.93	4.78-5.08
2012	4.77	4.67-4.87	4.31	4.18-4.45	5.26	5.10-5.41
2013	4.69	4.58-4.80	4.17	4.04-4.32	5.24	5.08-5.41
2014	4.80	4.69-4.92	4.21	4.06-4.36	5.44	5.27-5.62
2015	5.07	4.94-5.20	4.55	4.38-4.73	5.62	5.42-5.82
2016	4.89	4.74-5.05	4.44	4.24-4.65	5.38	5.15-5.61
2017	4.77	4.60-4.95	4.02	3.81-4.25	5.56	5.30-5.84
2018	4.58	4.40-4.78	3.86	3.63-4.11	5.34	5.06-5.64
Modelled rates						
	Both genders		Women		Men	
	Rate	95% CI	Rate	95% CI	Rate	95% CI
2004	4.44	4.34-4.54	4.14	4.01-4.28	4.75	4.60-4.90
2005	4.30	4.21-4.40	3.98	3.85-4.11	4.66	4.51-4.80
2006	4.47	4.38-4.57	4.14	4.01-4.27	4.84	4.69-4.98
2007	4.33	4.23-4.43	3.93	3.80-4.05	4.76	4.62-4.91
2008	4.40	4.30-4.49	4.11	3.98-4.24	4.71	4.56-4.85
2009	4.35	4.26-4.45	3.99	3.86-4.12	4.74	4.60-4.89
2010	4.27	4.18-4.37	3.83	3.71-3.96	4.75	4.60-4.90
2011	4.50	4.40-4.60	4.10	3.97-4.23	4.93	4.78-5.09
2012	4.75	4.65-4.86	4.33	4.19-4.47	5.21	5.05-5.37
2013	4.65	4.55-4.76	4.19	4.05-4.33	5.14	4.98-5.31
2014	4.72	4.61-4.84	4.19	4.04-4.34	5.29	5.11-5.46
2015	4.97	4.84-5.11	4.54	4.37-4.72	5.44	5.24-5.64
2016	4.84	4.69-5.00	4.48	4.28-4.69	5.23	5.00-5.47
2017	4.86	4.69-5.04	4.21	3.99-4.45	5.55	5.29-5.83
2018	4.66	4.47-4.85	4.05	3.81-4.30	5.31	5.02-5.60

For peer review only

Appendix 5: Observed and modelled incidence rates (per 1,000 person-years) for recorded AF during the period 2004-2018 – stratified by calendar year, age and gender.

Both genders – Observed rates										
	40-54		55-64		65-74		75-84		85+	
Year	Rate	95% CI	Rate	95% CI	Rate	95% CI	Rate	95% CI	Rate	95% CI
2004	0.56	0.51-0.62	2.33	2.19-2.48	6.99	6.69-7.29	15.1	14.6-15.7	20.0	19.0-21.2
2005	0.62	0.57-0.68	2.21	2.08-2.35	6.83	6.54-7.13	14.6	14.0-15.1	19.1	18.0-20.1
2006	0.54	0.49-0.59	2.43	2.29-2.58	6.98	6.69-7.28	15.4	14.9-16.0	20.0	19.0-21.1
2007	0.55	0.50-0.60	2.30	2.16-2.44	6.70	6.42-7.00	14.4	13.9-15.0	21.0	20.0-22.1
2008	0.54	0.49-0.59	2.41	2.28-2.56	6.86	6.57-7.15	14.8	14.3-15.4	20.4	19.4-21.5
2009	0.55	0.50-0.61	2.28	2.14-2.42	6.60	6.32-6.89	14.6	14.0-15.1	21.9	20.9-23.0
2010	0.51	0.46-0.56	2.24	2.11-2.39	6.49	6.21-6.79	14.8	14.3-15.4	20.4	19.4-21.5
2011	0.57	0.52-0.62	2.42	2.27-2.57	6.65	6.37-6.95	15.5	14.9-16.1	21.9	20.9-23.0
2012	0.57	0.52-0.63	2.42	2.27-2.58	7.27	6.97-7.58	16.5	15.9-17.1	22.8	21.7-23.9
2013	0.62	0.56-0.68	2.35	2.19-2.51	6.74	6.45-7.04	16.3	15.7-16.9	23.1	21.9-24.3
2014	0.62	0.56-0.69	2.38	2.21-2.55	6.86	6.55-7.19	16.7	16.0-17.4	23.2	22.0-24.4
2015	0.68	0.61-0.75	2.62	2.43-2.83	6.86	6.51-7.22	17.7	17.0-18.5	24.9	23.5-26.3
2016	0.64	0.56-0.73	2.41	2.20-2.65	7.18	6.76-7.63	16.5	15.6-17.4	24.9	23.2-26.6
2017	0.62	0.53-0.72	2.57	2.32-2.85	7.10	6.62-7.62	16.7	15.7-17.8	24.8	22.9-26.9
2018	0.63	0.54-0.75	2.37	2.11-2.66	6.97	6.45-7.53	15.7	14.7-16.9	23.7	21.7-26.0
Women – Observed rates										
	40-54		55-64		65-74		75-84		85+	
Year	Rate	95% CI	Rate	95% CI	Rate	95% CI	Rate	95% CI	Rate	95% CI
2004	0.293	0.243-0.354	1.52	1.36-1.69	5.61	5.25-5.99	13.9	13.2-14.6	19.1	17.9-20.5
2005	0.369	0.313-0.435	1.40	1.26-1.57	5.38	5.03-5.75	13.1	12.4-13.7	18.6	17.4-19.9
2006	0.319	0.267-0.380	1.64	1.48-1.81	5.70	5.34-6.09	13.6	13.0-14.3	18.7	17.6-20.0
2007	0.314	0.263-0.375	1.52	1.37-1.69	5.00	4.66-5.36	12.7	12.1-13.4	19.7	18.5-21.0
2008	0.293	0.245-0.352	1.61	1.46-1.79	5.45	5.10-5.82	13.5	12.8-14.2	19.7	18.5-21.0
2009	0.286	0.238-0.344	1.42	1.27-1.58	5.04	4.71-5.40	13.0	12.4-13.7	20.9	19.6-22.2
2010	0.283	0.234-0.341	1.50	1.35-1.67	4.87	4.54-5.22	12.8	12.2-13.5	18.4	17.3-19.7
2011	0.298	0.248-0.359	1.70	1.54-1.89	5.18	4.84-5.55	13.3	12.7-14.1	20.3	19.0-21.6
2012	0.306	0.255-0.368	1.49	1.33-1.67	5.56	5.21-5.94	14.6	13.8-15.3	21.5	20.2-22.9
2013	0.394	0.333-0.466	1.39	1.23-1.58	5.10	4.75-5.47	14.0	13.3-14.8	21.4	20.1-22.8
2014	0.340	0.280-0.412	1.48	1.30-1.68	4.79	4.43-5.17	14.8	14.0-15.6	21.0	19.6-22.4
2015	0.330	0.265-0.411	1.59	1.38-1.82	5.11	4.71-5.55	16.1	15.1-17.1	23.2	21.6-25.0
2016	0.339	0.262-0.439	1.61	1.37-1.89	5.73	5.22-6.29	14.3	13.2-15.4	23.6	21.7-25.8
2017	0.398	0.304-0.521	1.47	1.21-1.78	4.91	4.37-5.52	13.5	12.3-14.8	23.2	20.9-25.7
2018	0.309	0.221-0.433	1.56	1.28-1.91	4.83	4.25-5.49	13.2	11.9-14.6	21.5	19.1-24.2

For peer review only

Men – Observed rates										
	40-54		55-64		65-74		75-84		85+	
Year	Rate	95% CI	Rate	95% CI	Rate	95% CI	Rate	95% CI	Rate	95% CI
2004	0.824	0.738-0.920	3.13	2.91-3.38	8.52	8.06-9.02	17.0	16.1-17.9	22.2	20.2-24.5
2005	0.870	0.783-0.967	3.01	2.79-3.25	8.44	7.98-8.93	16.8	15.9-17.7	20.1	18.2-22.2
2006	0.750	0.669-0.839	3.22	2.99-3.46	8.40	7.93-8.88	17.9	17.0-18.9	23.1	21.1-25.3
2007	0.773	0.692-0.863	3.08	2.86-3.31	8.58	8.12-9.07	16.9	16.0-17.8	24.0	22.0-26.1
2008	0.770	0.689-0.859	3.21	2.99-3.45	8.41	7.95-8.89	16.7	15.9-17.6	22.1	20.2-24.1
2009	0.806	0.724-0.898	3.15	2.92-3.39	8.31	7.86-8.79	16.7	15.8-17.6	24.3	22.4-26.4
2010	0.725	0.647-0.813	2.99	2.77-3.23	8.28	7.83-8.76	17.5	16.6-18.4	24.8	22.9-27.0
2011	0.828	0.742-0.923	3.14	2.91-3.39	8.28	7.82-8.76	18.4	17.4-19.4	25.5	23.5-27.7
2012	0.834	0.748-0.931	3.36	3.12-3.63	9.15	8.67-9.66	19.1	18.2-20.1	25.4	23.4-27.6
2013	0.839	0.749-0.940	3.31	3.06-3.59	8.55	8.08-9.05	19.4	18.4-20.4	26.6	24.5-28.8
2014	0.901	0.802-1.013	3.28	3.01-3.58	9.15	8.63-9.70	19.2	18.2-20.3	27.6	25.4-30.1
2015	1.015	0.897-1.149	3.67	3.35-4.01	8.79	8.22-9.39	19.9	18.7-21.1	28.2	25.7-30.9
2016	0.927	0.795-1.082	3.22	2.87-3.60	8.79	8.12-9.52	19.4	18.0-21.0	27.3	24.4-30.5
2017	0.832	0.692-1.000	3.65	3.24-4.12	9.52	8.71-10.4	20.8	19.1-22.7	27.8	24.4-31.6
2018	0.952	0.788-1.150	3.17	2.76-3.65	9.32	8.46-10.3	19.1	17.3-21.0	27.6	24.0-31.8

Both genders – Modelled rates										
	40-54		55-64		65-74		75-84		85+	
Year	Rate	95% CI	Rate	95% CI	Rate	95% CI	Rate	95% CI	Rate	95% CI
2004	0.594	0.540-0.652	2.38	2.24-2.54	6.76	6.47-7.06	15.1	14.5-15.6	20.8	19.7-22.0
2005	0.604	0.550-0.661	2.38	2.24-2.52	6.61	6.32-6.91	14.5	14.0-15.0	19.6	18.6-20.7
2006	0.588	0.536-0.644	2.39	2.25-2.54	6.85	6.56-7.15	15.3	14.7-15.8	20.8	19.7-21.9
2007	0.558	0.507-0.612	2.28	2.14-2.42	6.57	6.28-6.86	14.8	14.3-15.4	20.6	19.6-21.7
2008	0.586	0.534-0.641	2.35	2.22-2.50	6.69	6.40-6.98	15.0	14.4-15.5	20.7	19.7-21.7
2009	0.550	0.500-0.604	2.25	2.12-2.40	6.53	6.25-6.82	15.0	14.4-15.5	21.3	20.3-22.4
2010	0.528	0.479-0.581	2.20	2.07-2.35	6.47	6.19-6.76	14.8	14.2-15.3	20.5	19.5-21.6
2011	0.565	0.513-0.620	2.34	2.19-2.49	6.82	6.52-7.12	15.5	14.9-16.1	21.6	20.5-22.7
2012	0.595	0.541-0.653	2.45	2.30-2.61	7.15	6.85-7.45	16.4	15.8-17.0	23.2	22.1-24.3
2013	0.570	0.515-0.628	2.38	2.22-2.54	6.99	6.69-7.30	16.1	15.5-16.7	22.7	21.6-23.9
2014	0.581	0.523-0.645	2.42	2.25-2.59	7.09	6.77-7.42	16.3	15.7-17.0	23.1	21.9-24.4
2015	0.621	0.553-0.695	2.55	2.35-2.75	7.42	7.06-7.80	17.1	16.4-17.9	24.7	23.3-26.1
2016	0.589	0.511-0.676	2.45	2.23-2.69	7.24	6.81-7.69	16.8	15.9-17.7	24.0	22.3-25.7
2017	0.595	0.506-0.694	2.48	2.23-2.76	7.30	6.81-7.83	16.8	15.8-17.9	23.8	21.9-25.8

For peer review only - http://bmjopen.bmj.com/site/about/guidelines.xhtml

2018	0.585	0.489-0.694	2.41	2.14-2.70	7.02	6.49-7.59	16.1	15.0-17.2	22.6	20.5-24.7

Women – Modelled rates										
	40-54		55-64		65-74		75-84		85+	
Year	Rate	95% CI	Rate	95% CI	Rate	95% CI	Rate	95% CI	Rate	95% CI
2004	0.350	0.292-0.416	1.63	1.46-1.81	5.30	4.95-5.67	13.4	12.8-14.1	20.5	19.2-21.8
2005	0.350	0.293-0.415	1.60	1.44-1.78	5.14	4.79-5.50	12.8	12.2-13.5	19.3	18.0-20.5
2006	0.378	0.319-0.445	1.71	1.54-1.88	5.40	5.04-5.77	13.3	12.7-14.0	19.7	18.5-21.0
2007	0.322	0.268-0.383	1.51	1.35-1.68	4.97	4.63-5.33	12.8	12.1-13.4	19.7	18.5-21.0
2008	0.346	0.291-0.409	1.61	1.45-1.78	5.25	4.91-5.62	13.3	12.7-14.0	20.3	19.1-21.6
2009	0.296	0.245-0.355	1.44	1.29-1.60	4.92	4.58-5.27	13.1	12.4-13.8	20.9	19.7-22.2
2010	0.318	0.265-0.380	1.48	1.33-1.65	4.87	4.54-5.22	12.4	11.8-13.1	19.1	17.9-20.4
2011	0.340	0.284-0.404	1.59	1.42-1.77	5.22	4.87-5.59	13.3	12.6-14.0	20.4	19.1-21.7
2012	0.330	0.274-0.394	1.59	1.42-1.78	5.38	5.03-5.75	14.2	13.4-14.9	22.4	21.1-23.8
2013	0.325	0.268-0.391	1.55	1.38-1.74	5.23	4.87-5.60	13.7	12.9-14.5	21.5	20.2-23.0
2014	0.313	0.253-0.383	1.52	1.33-1.72	5.17	4.79-5.57	13.7	12.9-14.6	21.9	20.5-23.5
2015	0.318	0.251-0.397	1.58	1.37-1.81	5.51	5.08-5.97	14.9	14.0-15.9	24.3	22.6-26.1
2016	0.339	0.258-0.439	1.64	1.39-1.92	5.55	5.04-6.10	14.7	13.6-15.8	23.3	21.3-25.4
2017	0.308	0.221-0.418	1.51	1.24-1.83	5.18	4.60-5.80	13.8	12.6-15.2	22.2	19.9-24.6
2018	0.300	0.207-0.422	1.46	1.17-1.80	5.00	4.38-5.67	13.3	11.9-14.7	21.1	18.7-23.8

Men – Modelled rates										
	40-54		55-64		65-74		75-84		85+	
Year	Rate	95% CI	Rate	95% CI	Rate	95% CI	Rate	95% CI	Rate	95% CI
2004	0.829	0.740-0.925	3.14	2.91-3.39	8.37	7.90-8.86	17.3	16.4-18.3	21.6	19.5-23.8
2005	0.847	0.759-0.943	3.15	2.92-3.40	8.24	7.77-8.72	16.8	15.9-17.7	20.5	18.5-22.6
2006	0.792	0.707-0.884	3.08	2.86-3.32	8.45	7.98-8.94	18.0	17.1-19.0	23.1	21.0-25.2
2007	0.787	0.703-0.878	3.06	2.83-3.29	8.33	7.87-8.82	17.7	16.8-18.6	22.6	20.6-24.7
2008	0.816	0.731-0.908	3.10	2.87-3.33	8.28	7.82-8.76	17.2	16.3-18.1	21.5	19.6-23.5
2009	0.797	0.713-0.888	3.07	2.84-3.30	8.31	7.85-8.79	17.5	16.6-18.5	22.2	20.3-24.3
2010	0.732	0.652-0.821	2.93	2.71-3.17	8.23	7.77-8.71	18.0	17.1-19.0	23.7	21.8-25.8
2011	0.782	0.696-0.874	3.09	2.85-3.34	8.58	8.11-9.08	18.6	17.6-19.6	24.1	22.2-26.3
2012	0.850	0.760-0.947	3.31	3.06-3.58	9.10	8.61-9.60	19.4	18.4-20.4	25.0	22.9-27.1
2013	0.806	0.715-0.905	3.20	2.94-3.47	8.94	8.44-9.45	19.4	18.4-20.5	25.4	23.3-27.6
2014	0.843	0.744-0.952	3.32	3.04-3.62	9.21	8.68-9.76	19.9	18.8-21.0	25.8	23.6-28.1
2015	0.910	0.795-1.037	3.52	3.20-3.86	9.53	8.93-10.16	20.1	18.9-21.4	25.5	23.1-28.2
2016	0.829	0.699-0.976	3.28	2.92-3.67	9.11	8.41-9.84	19.7	18.3-21.2	25.6	22.7-28.7
2017	0.876	0.725-1.048	3.46	3.04-3.92	9.65	8.82-10.53	21.0	19.2-22.8	27.4	24.0-31.2
2018	0.854	0.692-1.043	3.35	2.91-3.84	9.25	8.37-10.20	19.9	18.0-21.9	25.7	22.1-29.7

For peer review only

Appendix 6: Modelled incidence of recorded AF over time by patient deprivation (most vs least deprived quintiles of IMD.

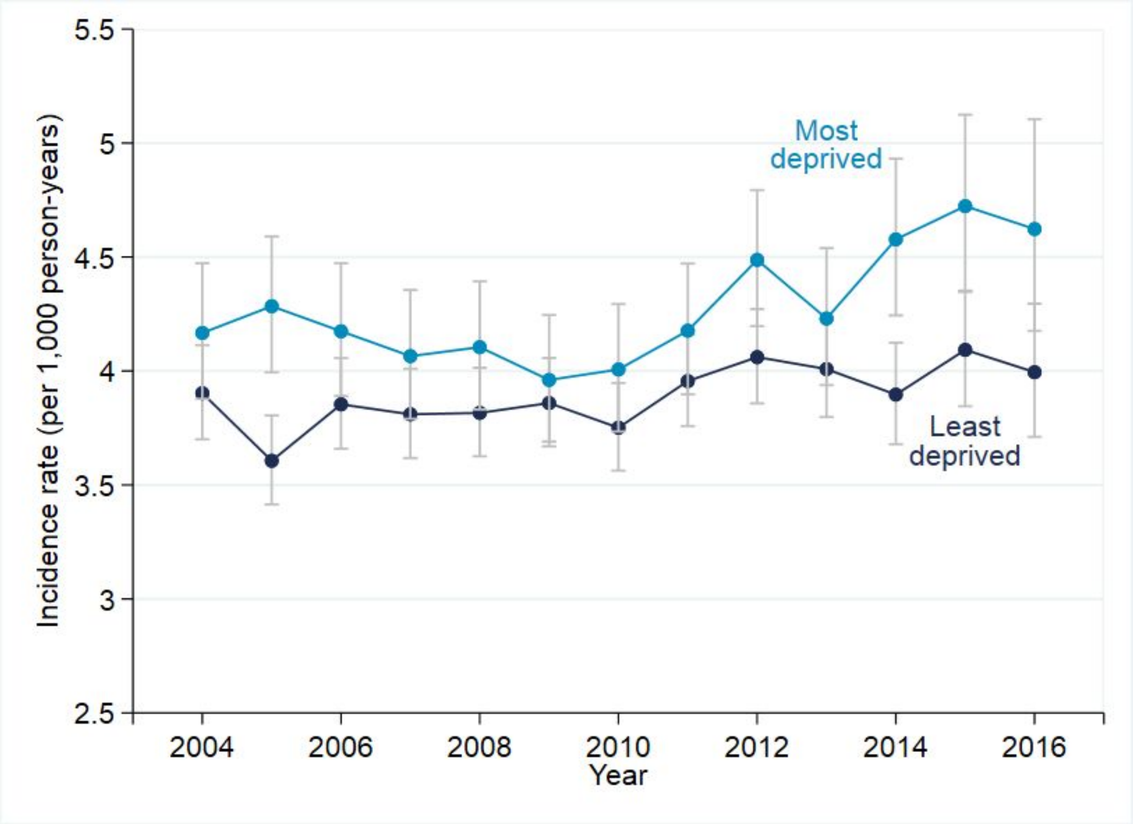

For peer review only - http://bmjopen.bmj.com/site/about/guidelines.xhtml

Appendix 7: Model results for the association between deprivation and incidence rates.

	IRR (95% CI)	p
IMD (ref=1, least deprived)		0.002
2	1.06 (1.02-1.10)	
3	1.07 (1.01-1.14)	
4	1.10 (1.01-1.20)	
5, most deprived	1.17 (1.05-1.31)	
c.IMD*i.year (ref=2016)		0.182
2004	0.98 (0.95-1.01)	
2005	1.01 (0.97-1.04)	
2006	0.98 (0.95-1.02)	
2007	0.98 (0.95-1.01)	
2008	0.98 (0.95-1.02)	
2009	0.97 (0.94-1.00)	
2010	0.98 (0.95-1.01)	
2011	0.98 (0.95-1.01)	
2012	0.99 (0.96-1.02)	
2013	0.98 (0.95-1.01)	
2014	1.00 (0.97-1.04)	
2015	1.00 (0.97-1.04)	

Notes:

1) The model structure is as follows (as described in the methods section):
Year + agecat + gender + IMD + i.year*c.agecat + i.year*i.gender + i.year*c.IMD + c.agecat*i.gender + i.year#c.agecat*i.gender

2) This analysis uses a subsample of patients with complete IMD during the period 2004 to 2016. It includes a total of 74,514 AF incident cases and 17.8 million person-years of observation. Due to methodological differences between the IMD stratified denominator provided by CPRD and the way we have calculated the incidence rates, the rate for all patients in this subsample is slightly lower than our main analysis rate. Therefore we prefer to show relative differences (as incidence rate ratios, IRR) instead of absolute differences in the quintiles of IMD.

For peer review only - http://bmjopen.bmj.com/site/about/guidelines.xhtml

Appendix 8: Observed incidence rates (per 1,000 person-years) for recorded AF during the period 2004-2018. This is an additional stratification by age (under and over 65), for comparison with other studies.

	Incidence rate	95% CI
40-64	1.21	1.19-1.23
65+	11.6	11.5-11.6
Women		
40-64	0.75	0.73-0.77
65+	10.4	10.3-10.5
Men		
40-64	1.66	1.63-1.69
65+	13.0	12.9-13.2

For peer review only

For peer review only - http://bmjopen.bmj.com/site/about/guidelines.xhtml

The RECORD statement – checklist of items, extended from the STROBE statement, that should be reported in observational studies using routinely collected health data.

	Item No.	STROBE items	Location in manuscript where items are reported	RECORD items	Location in manuscript where items are reported
**Title and abstract**					
	1	(a) Indicate the study's design with a commonly used term in the title or the abstract (b) Provide in the abstract an informative and balanced summary of what was done and what was found		RECORD 1.1: The type of data used should be specified in the title or abstract. When possible, the name of the databases used should be included.<br><br>RECORD 1.2: If applicable, the geographic region and timeframe within which the study took place should be reported in the title or abstract.<br><br>RECORD 1.3: If linkage between databases was conducted for the study, this should be clearly stated in the title or abstract.	Abstract –setting<br><br>Abstract – objective and participants<br><br>Abstract - Setting
**Introduction**					
Background rationale	2	Explain the scientific background and rationale for the investigation being reported	Introduction – sentences 4 to 10		
Objectives	3	State specific objectives, including any prespecified hypotheses	Last sentence of introduction		
**Methods**					
Study Design	4	Present key elements of study design early in the paper	Methods – Data source and Study cohort and disease definition		
Setting	5	Describe the setting, locations, and relevant dates, including	Methods –Study cohort and disease definition		

For peer review only

		periods of recruitment, exposure, follow-up, and data collection			
Participants	6	*(a) Cohort study* - Give the eligibility criteria, and the sources and methods of selection of participants. Describe methods of follow-up<br>*Case-control study* - Give the eligibility criteria, and the sources and methods of case ascertainment and control selection. Give the rationale for the choice of cases and controls<br>*Cross-sectional study* - Give the eligibility criteria, and the sources and methods of selection of participants<br><br>*(b) Cohort study* - For matched studies, give matching criteria and number of exposed and unexposed<br>*Case-control study* - For matched studies, give matching criteria and the number of controls per case		RECORD 6.1: The methods of study population selection (such as codes or algorithms used to identify subjects) should be listed in detail. If this is not possible, an explanation should be provided.<br><br>RECORD 6.2: Any validation studies of the codes or algorithms used to select the population should be referenced. If validation was conducted for this study and not published elsewhere, detailed methods and results should be provided.<br><br>RECORD 6.3: If the study involved linkage of databases, consider use of a flow diagram or other graphical display to demonstrate the data linkage process, including the number of individuals with linked data at each stage.	Methods - Study cohort and disease definition. Codes used to identify AF cases provided in Appendix 2.<br><br>Methods - Study cohort and disease definition references where our codelist was adapted from.<br><br>The flowchart in Appendix 3 shows how we identified patients contributing to the numerator/denominator.
Variables	7	Clearly define all outcomes, exposures, predictors, potential confounders, and effect modifiers. Give diagnostic criteria, if applicable.		RECORD 7.1: A complete list of codes and algorithms used to classify exposures, outcomes, confounders, and effect modifiers should be provided. If these cannot be reported, an explanation should be provided.	Codes used to identify AF cases are provided in Appendix 2.
Data sources/ measurement	8	For each variable of interest, give sources of data and details of methods of assessment (measurement).	In Methods – Data analysis we provide a detailed explanation for how eligible time of follow up and AF		

For peer review only

		Describe comparability of assessment methods if there is more than one group	cases were determined – paragraphs 1 and 2, respectively.		
Bias	9	Describe any efforts to address potential sources of bias	In Methods – Data analysis paragraph 6 we explain the approach taken to address a potential source of bias.		
Study size	10	Explain how the study size was arrived at	The flowchart in Appendix 3 shows how we arrived at the final numbers of patients contributing to the numerator/denominator.		
Quantitative variables	11	Explain how quantitative variables were handled in the analyses. If applicable, describe which groupings were chosen, and why	In Methods – Data analysis paragraph 4 we explain how we categorized age.		
Statistical methods	12	(a) Describe all statistical methods, including those used to control for confounding<br>(b) Describe any methods used to examine subgroups and interactions<br>(c) Explain how missing data were addressed<br>(d) *Cohort study* - If applicable, explain how loss to follow-up was addressed<br>*Case-control study* - If applicable, explain how matching of cases and controls was addressed	(a) We used stratification and modelling which were described in paragraphs 5 and 6 of Methods – Data analysis.<br>(b) The modelling approach included interaction terms described in paragraph 6 of Data analysis.<br>(c) This study focuses on recorded		

For peer review only

		Cross-sectional study - If applicable, describe analytical methods taking account of sampling strategy<br>(e) Describe any sensitivity analyses	AF so it doesn't include patients whose diagnosis is not recorded in their medical records – this is clarified in the Strengths & Limitation section, sentence 2.<br>(d) only time during eligible follow-up is included in the analysis; this is described in paragraph 1 of Data Analysis.<br>(e) Not applicable		
Data access and cleaning methods		..		RECORD 12.1: Authors should describe the extent to which the investigators had access to the database population used to create the study population.<br><br>RECORD 12.2: Authors should provide information on the data cleaning methods used in the study.	The information we used to identify cases is explained in methods-data analysis paragraphs 1 and 2.<br>This is an incidence study that did not involve a lot of data cleaning. We explain how we handled events after death in methods-data analysis paragraph 2.

For peer review only

Linkage		..		RECORD 12.3: State whether the study included person-level, institutional-level, or other data linkage across two or more databases. The methods of linkage and methods of linkage quality evaluation should be provided.	Included in Methods – Data analysis paragraph 7. A link to details on the IMD-linkage is available in the data sharing statement.
**Results**					
Participants	13	(a) Report the numbers of individuals at each stage of the study (*e.g.*, numbers potentially eligible, examined for eligibility, confirmed eligible, included in the study, completing follow-up, and analysed)<br>(b) Give reasons for non-participation at each stage.<br>(c) Consider use of a flow diagram		RECORD 13.1: Describe in detail the selection of the persons included in the study (*i.e.,* study population selection) including filtering based on data quality, data availability and linkage. The selection of included persons can be described in the text and/or by means of the study flow diagram.	Flowchart for selected patients shown in Appendix 3. Final numbers reported in Results paragraph 1.
Descriptive data	14	(a) Give characteristics of study participants (*e.g.*, demographic, clinical, social) and information on exposures and potential confounders<br>(b) Indicate the number of participants with missing data for each variable of interest<br>(c) *Cohort study* - summarise follow-up time (*e.g.*, average and total amount)	(a)Patient characteristics reported in results section paragraph 1 and Table 1.<br>(b) Participants excluded because of missing data for gender are shown in Appendix 3.<br>(c) Total follow up time reported in the first sentence of Results section.		
Outcome data	15	*Cohort study* - Report numbers of outcome events or summary measures over time	Number of new AF cases in the study period is reported in		

For peer review only

		Case-control study - Report numbers in each exposure category, or summary measures of exposure<br>*Cross-sectional study* - Report numbers of outcome events or summary measures	the first sentence of Results section.		
Main results	16	(a) Give unadjusted estimates and, if applicable, confounder-adjusted estimates and their precision (e.g., 95% confidence interval). Make clear which confounders were adjusted for and why they were included<br>(b) Report category boundaries when continuous variables were categorized<br>(c) If relevant, consider translating estimates of relative risk into absolute risk for a meaningful time period	(a) Observed rates stratified by age and gender are shown in Table 2. Modelled (adjusted) rates are shown in Figures 1 and 2 and appendix 6. Both observed and modelled rates are included in Appendices 4 and 5.<br>(b) Not applicable<br>(c) Our main analysis presents absolute risk estimates as incidence rates.		
Other analyses	17	Report other analyses done—e.g., analyses of subgroups and interactions, and sensitivity analyses	One additional analysis including deprivation is included as Appendix 6 and 7.		
**Discussion**					
Key results	18	Summarise key results with reference to study objectives	Shown in Discussion - Summary		
Limitations	19	Discuss limitations of the study, taking into account sources of potential bias or imprecision. Discuss both direction and magnitude of any potential bias		RECORD 19.1: Discuss the implications of using data that were not created or collected to answer the specific research question(s). Include discussion of misclassification bias, unmeasured confounding, missing	Included in Discussion - Strengths and limitations

For peer review only

				data, and changing eligibility over time, as they pertain to the study being reported.	
Interpretation	20	Give a cautious overall interpretation of results considering objectives, limitations, multiplicity of analyses, results from similar studies, and other relevant evidence	Included in Discussion – Implication for research, policy and practice paragraph 1.		
Generalisability	21	Discuss the generalisability (external validity) of the study results	Included in Discussion - comparison with existing literature.		
**Other Information**					
Funding	22	Give the source of funding and the role of the funders for the present study and, if applicable, for the original study on which the present article is based	Included in Funding statement.		
Accessibility of protocol, raw data, and programming code		..		RECORD 22.1: Authors should provide information on how to access any supplemental information such as the study protocol, raw data, or programming code.	Included in the Data sharing statement.

*Reference: Benchimol EI, Smeeth L, Guttmann A, Harron K, Moher D, Petersen I, Sørensen HT, von Elm E, Langan SM, the RECORD Working Committee. The REporting of studies Conducted using Observational Routinely-collected health Data (RECORD) Statement. *PLoS Medicine* 2015; in press.

*Checklist is protected under Creative Commons Attribution (CC BY) license.

For peer review only

Temporal trends in incidence of atrial fibrillation in primary care records: a population-based cohort study

Journal:	*BMJ Open*
Manuscript ID	bmjopen-2020-042518.R1
Article Type:	Original research
Date Submitted by the Author:	05-Nov-2020
Complete List of Authors:	Mendonca, Silvia; University of Cambridge, Primary Care Unit, Department of Public Health & Primary Care<br>Saunders, Catherine; University of Cambridge, Primary Care Unit, Department of Public Health & Primary Care<br>Lund, Jenny; University of Cambridge, Primary Care Unit, Department of Public Health & Primary Care<br>Mant, Jonathan; University of Cambridge, Primary Care Unit, Department of Public Health & Primary Care<br>Edwards, Duncan; University of Cambridge, Primary Care Unit, Department of Public Health & Primary Care
**Primary Subject Heading**:	General practice / Family practice
Secondary Subject Heading:	Epidemiology, Health services research
Keywords:	PRIMARY CARE, EPIDEMIOLOGY, Health policy < HEALTH SERVICES ADMINISTRATION & MANAGEMENT

SCHOLARONE™ Manuscripts

I, the Submitting Author has the right to grant and does grant on behalf of all authors of the Work (as defined in the below author licence), an exclusive licence and/or a non-exclusive licence for contributions from authors who are: i) UK Crown employees; ii) where BMJ has agreed a CC-BY licence shall apply, and/or iii) in accordance with the terms applicable for US Federal Government officers or employees acting as part of their official duties; on a worldwide, perpetual, irrevocable, royalty-free basis to BMJ Publishing Group Ltd ("BMJ") its licensees and where the relevant Journal is co-owned by BMJ to the co-owners of the Journal, to publish the Work in this journal and any other BMJ products and to exploit all rights, as set out in our licence.

The Submitting Author accepts and understands that any supply made under these terms is made by BMJ to the Submitting Author unless you are acting as an employee on behalf of your employer or a postgraduate student of an affiliated institution which is paying any applicable article publishing charge ("APC") for Open Access articles. Where the Submitting Author wishes to make the Work available on an Open Access basis (and intends to pay the relevant APC), the terms of reuse of such Open Access shall be governed by a Creative Commons licence – details of these licences and which Creative Commons licence will apply to this Work are set out in our licence referred to above.

Other than as permitted in any relevant BMJ Author's Self Archiving Policies, I confirm this Work has not been accepted for publication elsewhere, is not being considered for publication elsewhere and does not duplicate material already published. I confirm all authors consent to publication of this Work and authorise the granting of this licence.

For peer review only

Title: Temporal trends in incidence of atrial fibrillation in primary care records: a population-based cohort study

Co-authors: Sílvia C Mendonça, Catherine L Saunders, Jenny Lund, Jonathan Mant, Duncan Edwards

Primary Care Unit, Department of Public Health and Primary Care, University of Cambridge, Institute of Public Health, Forvie Site, Robinson Way, Cambridge, CB2 0SR

Correspondence: Silvia Mendonca

Institute of Public Health, Forvie Site, Robinson Way, Cambridge, CB2 0SR

sm2061@medschl.cam.ac.uk

For peer review only

ABSTRACT

Objectives Atrial fibrillation (AF) is a heart condition associated with a fivefold increased risk of stroke. The condition can be detected in primary care and treatment can greatly reduce the risk of stroke. In recent years, a number of policy initiatives have tried to improve diagnosis and treatment of AF, including local National Health Service (NHS) schemes and the Quality and Outcomes Framework. We aimed to examine trends in the incidence of recorded AF in primary care records from English practices between 2004 and 2018.

Design Longitudinal cohort study.

Setting English primary care electronic health records linked to Index of Multiple Deprivation data.

Participants Cohort of 3.5 million patients over 40 years old registered in general practices in England, contributing 22 million person-years of observation between 2004 and 2018.

Primary and secondary outcome measures Incident AF was identified through newly recorded AF codes in the patients' records. Yearly incidence rates were stratified by gender, age group and a measure of deprivation.

Results Incidence rates were stable before 2010 and then rose and peaked in 2015 at 5.1 (95% CI 4.94-5.20) cases per 1,000 person-years. Incidence was higher in males (4.95 [95% CI 4.91-4.99] cases per 1,000 person-years vs 4.12 [95% CI 4.08-4.16] in females) and rises markedly with age (0.58 [95% CI 0.56-0.59] cases per 1,000 person-years in 40-54 years old vs 21.7 [95% CI 21.4-22.0] cases in over 85s). The increase in incidence over time was observed mainly in people over the age of 75, particularly men. There was no evidence that incidence was associated with deprivation.

Conclusions Changes in clinical practice and policy initiatives since 2004 have been associated with increased rates of diagnosis of AF up until 2015, but rates declined from 2015 to 2018.

STRENGTHS AND LIMITATIONS OF THIS STUDY

- Our population based sample covered 3.5 million patients at risk over a period of 15 years.
- The data from the Clinical Practice Research Datalink (CPRD) used in this study have been shown to be representative of the UK population and to be of high quality for the recording of diagnoses, particularly for chronic conditions.
- Atrial fibrillation has been part of the Quality and Outcomes Framework since 2007 and is likely to be well recorded in primary care records.
- A limitation is that these reported incidence rates exclude patients with undiagnosed AF, or that don't have their diagnosis recorded in their primary care records.

INTRODUCTION

Atrial fibrillation is a cardiac arrhythmia that is associated with a five-fold increase in risk of stroke[1]. The risk of stroke associated with AF can be greatly reduced by use of anticoagulants. AF is associated with 1 in 5 stroke cases[2] and is considered to be underdiagnosed[3] and undertreated[4]. Consequently, AF has been the target of a number of policy initiatives in recent years aimed at both treatment and diagnosis. In the UK, it has been part of the Quality and Outcomes Framework (QOF) since 2006[5], the pay for performance scheme that incentivizes the recording and appropriate management of chronic conditions. Since 2008 there have been several local National Health Service (NHS) schemes to incentivize detection and management of AF including the GRASP-AF (Guidance on Risk Assessment and Stroke Prevention for Atrial Fibrillation) electronic tool[6] and opportunistic screening by manual pulse checks[5]. It is the subject of a national programme co-ordinated by the Academic Health Sciences Network[2]. The need for better detection is highlighted in the NHS Long Term Plan (2019)[7]. There is evidence from both UK and international studies of a rise in the age-specific incidence of AF[8,9], including two using UK electronic primary care records[10,11]. However, these have not used data beyond 2011. Given that policy initiatives continue to focus on AF, our aim was to describe temporal trends in recorded AF in a nationally representative sample of primary care records up until 2018.

METHODS

Data source

We used data from the Clinical Practice Research Datalink (CPRD) GOLD, a database of electronic primary care records in the UK, based on patients attending GP practices which use the Vision computer system. CPRD has been shown to be nationally representative of the UK population[12] and data quality is monitored at the practice and patient level. In 2013 it contained the medical records of over 11 million patients, with 4.4 active (currently registered) patients corresponding to about 7% of the UK population[12]. During the study period (2004 to 2018) the number of UK practices contributing data to CPRD varied from 680 to 367 (Appendix 1) due to a reduction of practices using the Vision computer system. The data collected from CPRD include all clinical codes for medical diagnoses entered at consultations, referrals, tests and all prescriptions issued at the practice.

For peer review only

For peer review only - http://bmjopen.bmj.com/site/about/guidelines.xhtml

Study cohort and disease definition

The study population included patients from English practices aged over 40 years old between the 1st January 2004 and 31st December 2018. Recorded AF was identified using a list of 8 Read codes (Appendix 2). This list was developed as part of a project on the epidemiology of multimorbidity[13,14] and was based originally on the QOF business rules. We used the October 2019 data release of CPRD GOLD.

Data analysis

AF incidence was estimated as the number of new AF cases during each year, divided by the eligible follow up (person-time at risk). Eligible follow up for all patients (with and without AF) was calculated using the CPRD denominator files, which contain basic information about each patient and practice. Start of follow up was calculated as the latest of: one year after the patient first registered at the practice, the practice up-to-standard date (an internal measure of CPRD data quality, based on consistency of data provided to CPRD by the practice[12]) and 1st of January 2004 (study start). The end of follow-up was the earliest of: the last date the patient was registered at the practice, date of death, the last date the practice contributed data to CPRD, or the 31st of December 2018. We restricted follow-up to start one year after registration at the practice to avoid capturing historical diagnosis.

People with a new AF medical code recorded for the first time during the eligible follow up period were identified as incident cases and counted towards the numerator. Those people with any AF code identified before the start of eligible follow up were considered prevalent cases and were excluded. Cases registered after the end of eligible follow up were also excluded from the numerator, except for a few cases that occurred within 3 months of death or transfer out date. We decided to include these as they probably relate to events that occurred within eligible follow up but that were registered later. AF cases that occurred within or after eligible follow up contribute time at risk to the denominator until their date of diagnosis or end of follow up, whichever is first.

Incidence rates are calculated as the number of new cases of AF in our eligible population and study period (numerator) divided by the total person time at risk (denominator). The denominator was the sum of the person-time at risk for all eligible patients (cases and non-cases).

The patients' sex and year of birth were available from CPRD. We assumed the patients' date of birth to be the mid-year (i.e. the 30th of June) of year of birth. Patients' age were grouped into 5 categories: 40 to 54, 55 to 64, 65 to 74, 75 to 84 and ≥85 years old.

Stratified incidence rates by calendar year and socio-demographic characteristics were calculated through the use of a Lexis expansion[15]. Each patient time at risk was split into multiple time periods according to their age group and calendar years. Time at risk is then added up within each strata, a combination of calendar year by age group by gender (eg, females aged 65 to 74 in 2016). The incidence rate corresponds to the number of AF cases divided by the total person-time at risk in that strata. P-values for the crude effects were derived from unadjusted poisson models; p-values for the effect of age and gender were calculated using a gender-stratified model and a model with the two-way interaction.

In order to take into account possible changes over time in the age composition of the sample we have additionally estimated modelled incidence rates. We used a poisson regression approach using the number of events in each strata as the main outcome and including the logarithm of follow

For peer review only - http://bmjopen.bmj.com/site/about/guidelines.xhtml

up time as an offset. The model for all patients included the main effect variables year, age group and sex and their two-way and three-way interactions. Year and sex were always treated as categorical variables but age group was treated as a continuous variable for the interaction terms only in order to maximize power. Each model was used to predict the incidence rate for each year adjusting for the case-mix characteristics of the whole sample (known as marginal standardization)[16].

Incidence by deprivation was calculated using a CPRD patient denominator file stratified by the patient level 2010 English Index of Multiple Deprivation (IMD) quintiles and the linked patient level IMD quintiles for the patients with newly registered AF. For this data linkage, data are only available until the end of 2016. To model the effect of deprivation we used the same model as the main analysis but adding in a categorical main effect for deprivation quintile and an interaction between year and deprivation quintile (treated as a linear term).

Data manipulation and analyses were performed in Stata (StataCorp, version 15.1). We report 95% confidence intervals calculated as exact Poisson confidence intervals.

Patient and Public Involvement

Patients and the public were not involved in this study.

RESULTS

In a study population of nearly 3.5 million patients contributing a total of 22 million person-years of observation, there were 99,836 newly registered AF cases (see patient selection flowchart in Appendix 3) with a mean age at diagnosis of 74.6 years (see Table 1). Over 55% of new diagnoses were in people over the age of 75. The overall incidence is 4.52 cases per 1,000 patient years in people aged over 40 years old, and 11.6 per 1,000 patient years in people aged 65 years and older. The incidence rises by age, up to 21.7 per 1,000 in people aged 85 or over, and is higher in men than women, 4.95 versus 4.12 per 1,000 person years (Table 2). There was evidence of significant variation associated with the effects of age and gender ($p<0.001$ for both, Table 2). We also noted that incidence increases with age differently for men and women ($p<0.001$, table 2 and figure 1).

Incidence of AF was relatively stable between 2004 and 2010, between 4.2 and 4.5 cases per 1,000 person-years, then increased up until 2015, when incidence peaked at 5.1 cases per 1,000 person-years, before falling back to 4.6 cases per 1,000 person years by 2018 (see figure 1 and appendix 4). There was strong evidence of variation between years ($p<0.001$). This post-2015 decline occurred primarily in women. The age-specific trends show that the increase between 2010 and 2015 occurred largely in people aged 75 and over, and more so in men (figure 2 and appendix 5).

Incidence of AF was higher in the most deprived quintile of IMD than in the least deprived quintile ($p=0.002$ for the effect of deprivation; Appendix 6 and 7). There was no evidence of differences in temporal trends in AF by levels of socio economic deprivation ($p=0.182$).

DISCUSSION

SUMMARY

We observed stable AF incidence from 2004 to 2010, increased incidence from 2010 to 2015, and then falling incidence 2016 to 2018. The rise in incidence was primarily in people aged 75 and

For peer review only - http://bmjopen.bmj.com/site/about/guidelines.xhtml

over and most marked in men. The decline from 2016 to 2018 reflected falling incidence in women rather than men.

STRENGTHS AND LIMITATIONS

A strength of this study was the use of a large sample that is representative of the English population, permitting a detailed description of current practice. It is important to note that the focus of this study was *recorded* (or clinically detected) atrial fibrillation, and thus patients with undiagnosed, or who are diagnosed but not recorded will not be included. However, diagnosis and inclusion in population registers is essential to ensure adequate care and stroke prevention, and the focus of this study was to describe the incidence of clinically detected AF. Our modelled incidence rates remove the effect that possible changes in the population case-mix over time could have on observed rates. However, changes in the number of practices using the Vision computer system in recent years could be contributing to the levelling off in incidence rates observed after 2015[17].

COMPARISON WITH EXISTING LITERATURE

Incidence rates for AF can vary considerably between different studies, most likely due to different source populations, case definitions and study periods. The rates reported in this study are similar to those described in other European cohorts from Iceland[18] and the Netherlands[19] but lower than one from Germany[20]. In general, incidence rates reported from American cohorts[21-23] are higher than those from Europe, including our own study.

In the UK population, we found two studies that looked at incidence of AF over time[10,11]. Scowcroft et al. calculated incidence rates between 2000 and 2012 while Lane et al. reports incidence rates between 1998 and 2010. Both studies use data from CPRD and also report increases in incidence (Scowcroft from 2009 onwards and Lane between the periods 1998-2001 and 2002-2006.) We found higher incidence rates than those reported by Lane and Scowcroft. Whereas we restricted our analysis to people aged 40 years and over, they both reported incidence in people aged 18 years and over. However, even when looking at comparable age groups, our rates were higher. For example, Lane et al reported an incidence rate of 3.26 per 1,000 people aged 65-74 between 2007 and 2010, whereas the observed incidence rate in our study in this age group was 6.84 per 1,000. This is likely to reflect analytical differences in how the denominator population was defined. In our study, we derived both the numerator and denominator by first defining the eligible follow up period for all patients and then identifying which AF cases fell in this eligibility period. In the other two UK studies these figures appear to have been derived separately, with stricter criteria applied to the cases (eg. Scowcroft et al. imposes a minimum of one year both pre and post diagnosis) than to the denominator, resulting in lower rates, though the observed temporal trends will not have been affected. We believe our estimates are truer reflections of the incidence of AF. A recent meta-analysis of AF screening studies in a general population estimated a detection rate of 1.44% in patients aged 65 and over[24]. This is slightly higher than our estimate of 1.16 cases per 100 person years in the comparable age group (see Appendix 8), which is what would be expected given that these were screening studies, whereas ours would have identified only those picked up from active case finding[3]. Further corroborative evidence that our incidence rates are more likely to be correct comes from the Framingham study, which reported incidence rates of 13.4 per 1,000 in men over the age of 50, and 8.6 per 1,000 in women. The higher rates in Framingham, despite the slightly younger age group, reflect the multiple sources of ascertainment that were used to identify AF. Detection rates from routine practice will be lower than those.

For peer review only - http://bmjopen.bmj.com/site/about/guidelines.xhtml

Ours is the first study to demonstrate a decline in the incidence of recorded AF between 2015-2018 in the UK, after taking account of changes over time in age composition of the population. Given that epidemiological studies such as Framingham have reported increases rather than decreases in AF incidence [9], this is likely to reflect a change in ascertainment in recent years.

Box 1: Key changes in policy and practice during the study period, 2004 to 2018.

Policy or Evidence Change	Year
Requirement for practice AF registers incorporated within QOF[25]	2007
First major NOAC RCT demonstrates effectiveness (RE-LY study)[26]	2009
Widespread introduction of local incentive schemes (LES) to diagnose and/or treat AF[27]	2011
Expansion of AF within QOF from three to four indicators[25]	2013
NICE guidance suggests a larger proportion of AF patients are eligible for anticoagulation[28]	2014

QOF, Quality and Outcomes Framework; NOAC**,** Novel oral anticoagulants; RCT, randomized controlled trial; NICE, National Institute for Health and Care Excellence.

IMPLICATIONS FOR RESEARCH, POLICY AND PRACTICE

Our results suggest that initiatives in policy and clinical practice since 2004 have been associated with increased rates of diagnosis of AF (Box 1). Although the increase in incidence seen in over 85 year olds may be explained in part by the increase mean age of this group over time, it is also plausible that initiatives to encourage pulse palpation in over 65s[5], or improved perceived tolerability of newer anticoagulants[29] have influenced clinicians to increase efforts to diagnose older patients. It is also notable that increases in AF diagnosis do not appear to have come at the expense of the widening social inequality that has been of particular concern in conditions such as cancer[30]. The small decline in AF incidence since 2015 may reflect reducing policy emphasis or a decline in the primary care workforce available to detect AF[31]. The decline in incidence may also reflect that "catch up" diagnoses were made in the years prior to 2015, boosting apparent incidence during that period. Given the emergence of new technologies which allow self-diagnosis of atrial fibrillation, it is likely that the incidence of diagnosis of AF will rise again in the future[32]. Indeed, data from studies that have used implantable loop recorders to detect AF suggest that there may be substantial proportions of people with short episodes of undiagnosed atrial fibrillation [33].

It is important to continue to monitor trends in AF incidence to assess impact of new technologies and initiatives. Our finding that incidence of AF in the under 65 age group remained low and did not change during the study period suggests first that there has been no substantive shift in the clinical presentation of this group to primary care that has led to changes in AF incidence in this population. Policy initiatives such as incentives for opportunistic screening have not focused

For peer review only - http://bmjopen.bmj.com/site/about/guidelines.xhtml

on younger people with AF; our study would tend to support this approach as the numbers of people identified would be likely to be very low.

Our research has also highlighted the methodological point that researchers should ensure they use the correct denominators for studies of incidence in primary care database analyses as small changes in approaches can lead to substantial errors in published rates, with potentially large implications for policy decisions based on these estimates. We would recommend that policymakers base future modelling decisions and analyses on the rates presented in this, rather than previous studies.

CONCLUSION

The incidence of diagnosed AF increased in the UK between 2004-2015, with the greatest increase among patients aged over 85, but with a small decline between 2015-18. Trials of AF screening that are powered to detect impact on stroke rates will provide evidence on the extent to which efforts should be made to reverse this decline.[34]

Acknowledgements This study is based in part on data from the Clinical Practice Research Datalink obtained under licence from the UK Medicines and Healthcare products Regulatory Agency. The data is provided by patients and collected by the NHS as part of their care and support. The interpretation and conclusions contained in this study are those of the author/s alone. JM is an NIHR Senior Investigator.

Contributors DE and JM had the original idea for the study. DE, SM, CS, JL and JM designed the study. SM undertook data extraction and performed the analysis. SM and DE wrote the first draft of the paper, which was revised in collaboration with all authors. DE acts as guarantor.

Funding This report is independent research funded by the National Institute for Health Research (NIHR School for Primary Care Research, reference FR11/290). The views expressed in this publication are those of the authors and not necessarily those of the NHS, the National Institute for Health Research or the Department of Health.

Competing interests All authors report grants from the NIHR School of Primary Care Research, during the conduct of the study. JL reports grants from the Wellcome Trust and the National Institute for Health Research, outside the submitted work. JM is on an advisory board for BMS/Pfizer on an AF screening trial, and is chief investigator of an NIHR funded programme grant on screening for atrial fibrillation. DE is a co-investigator on this same NIHR programme.

Ethics approval This study was approved by the Independent Scientific Advisory Board for Medicines and Healthcare products Regulatory Agency research (protocol reference number 17_079R). No further ethics approval is required.

Data sharing statement The study uses data from the Clinical Practice Research Datalink (CPRD). CPRD does not allow the sharing of patient-level data. The data specification for the CPRD dataset is available at: https://www.cprd.com/sites/default/files/CPRD%20GOLD%20Full%20Data%20Specification%20v2.0_0.pdf Additional information on the IMD patient level linkage data is available at: https://cprd.com/sites/default/files/Documentation_SmallAreaData_Patient_set18_v2.7.pdf. The

For peer review only - http://bmjopen.bmj.com/site/about/guidelines.xhtml

code list used in this study is part of a set of lists made available at www.phpc.cam.ac.uk/pcu/cprd_cam/codelists/.

For peer review only

For peer review only - http://bmjopen.bmj.com/site/about/guidelines.xhtml

Table 1: Characteristics of patients with newly diagnosed AF in the period 2004 to 2018.

	N (%)
All patients	99836
Gender	
Men	52823 (52.9)
Women	47013 (47.1)
Age, mean (SD)	74.6 (11.2)
Age group	
40 - 54	5597 (5.6)
55 - 64	12598 (12.6)
65 - 74	26308 (26.4)
75 - 84	36215 (36.3)
85+	19118 (19.1)

Table 2: Observed incidence rates (per 1,000 person-years) for recorded AF during the period 2004 to 2018– stratified by age and gender.

	Incidence rate	95% CI	P-value(1)
All patients	4.52	4.49-4.55	
Gender			<0.001
Female	4.12	4.08-4.16	
Male	4.95	4.91-4.99	
Age group			<0.001
40 - 54	0.58	0.56-0.59	
55 - 64	2.36	2.32-2.40	
65 - 74	6.84	6.76-6.93	
75 - 84	15.5	15.3-15.7	
85+	21.7	21.4-22.0	
Gender and age group			<0.001
Women			<0.001
40 - 54	0.32	0.30-0.34	
55 - 64	1.52	1.48-1.57	
65 - 74	5.24	5.14-5.34	
75 - 84	13.7	13.5-13.9	
85+	20.3	20.0-20.7	
Men			<0.001
40 - 54	0.83	0.80-0.85	
55 - 64	3.20	3.14-3.27	
65 - 74	8.61	8.48-8.75	
75 - 84	18.0	17.7-18.3	
85+	24.8	24.2-25.3	

(1) The p-values presented in this table come from unadjusted tests of whether there is variation by age and sex (overall, top two sets of rows) and variation by age, stratified by sex (below, bottom two sets of rows). The interaction P-value, however has a slightly different

For peer review only - http://bmjopen.bmj.com/site/about/guidelines.xhtml

interpretation and presents a measure of the strength of evidence of whether the effect of age is different for men and women (middle row).

Figure 1: Modelled incidence of recorded AF over time.

Figure 2: Modelled incidence of recorded AF over time stratified by age group and gender.

For peer review only

REFERENCES

1. Wolf PA, Abbott RD, Kannel WB. Atrial fibrillation as an independent risk factor for stroke: the Framingham Study. Stroke 1991;22:983-8.
2. The AHSN Network (2019) Atrial Fibrillation. . at https://www.ahsnnetwork.com/about-academic-health-science-networks/national-programmes-priorities/atrial-fibrillation.)
3. Fitzmaurice DA, Hobbs FD, Jowett S, et al. Screening versus routine practice in detection of atrial fibrillation in patients aged 65 or over: cluster randomised controlled trial. BMJ 2007;335:383.
4. Nieuwlaat R, Capucci A, Lip GY, et al. Antithrombotic treatment in real-life atrial fibrillation patients: a report from the Euro Heart Survey on Atrial Fibrillation. Eur Heart J 2006;27:3018-26.
5. Cole J, Torabi P, Dostal I, Homer K, Robson J. Opportunistic pulse checks in primary care to improve recognition of atrial fibrillation: a retrospective analysis of electronic patient records. Br J Gen Pract 2018;68:e388-e93.
6. Quality and Outcomes Framework - 2006-07. (Accessed 18.01.2020, at https://digital.nhs.uk/data-and-information/publications/statistical/quality-and-outcomes-framework-achievement-data/quality-and-outcomes-framework-2006-07.)
7. The NHS Long Term Plan (2019). (Accessed 15/02/2020, at https://www.longtermplan.nhs.uk/.)
8. Chugh SS, Havmoeller R, Narayanan K, et al. Worldwide epidemiology of atrial fibrillation: a Global Burden of Disease 2010 Study. Circulation 2014;129:837-47.
9. Schnabel RB, Yin X, Gona P, et al. 50 year trends in atrial fibrillation prevalence, incidence, risk factors, and mortality in the Framingham Heart Study: a cohort study. Lancet 2015;386:154-62.
10. Scowcroft AC, Cowie MR. Atrial fibrillation: improvement in identification and stroke preventive therapy - data from the UK Clinical Practice Research Datalink, 2000-2012. Int J Cardiol 2014;171:169-73.
11. Lane DA, Skjoth F, Lip GYH, Larsen TB, Kotecha D. Temporal Trends in Incidence, Prevalence, and Mortality of Atrial Fibrillation in Primary Care. J Am Heart Assoc 2017;6.
12. Herrett E, Gallagher AM, Bhaskaran K, et al. Data Resource Profile: Clinical Practice Research Datalink (CPRD). Int J Epidemiol 2015;44:827-36.
13. Cassell A, Edwards D, Harshfield A, et al. The epidemiology of multimorbidity in primary care: a retrospective cohort study. Br J Gen Pract 2018;68:e245-e51.
14. Payne RA, Mendonca SC, Elliott MN, et al. Development and validation of the Cambridge Multimorbidity Score. CMAJ 2020;192:E107-E14.
15. Clayton D, Hills M. Statistical models in epidemiology. Oxford ; New York: Oxford University Press; 1993.
16. Muller CJ, MacLehose RF. Estimating predicted probabilities from logistic regression: different methods correspond to different target populations. Int J Epidemiol 2014;43:962-70.
17. Kontopantelis E, Stevens RJ, Helms PJ, Edwards D, Doran T, Ashcroft DM. Spatial distribution of clinical computer systems in primary care in England in 2016 and implications for primary care electronic medical record databases: a cross-sectional population study. BMJ Open 2018;8:e020738.
18. Stefansdottir H, Aspelund T, Gudnason V, Arnar DO. Trends in the incidence and prevalence of atrial fibrillation in Iceland and future projections. Europace 2011;13:1110-7.
19. Heeringa J, van der Kuip DA, Hofman A, et al. Prevalence, incidence and lifetime risk of atrial fibrillation: the Rotterdam study. Eur Heart J 2006;27:949-53.
20. Wilke T, Groth A, Mueller S, et al. Incidence and prevalence of atrial fibrillation: an analysis based on 8.3 million patients. Europace 2013;15:486-93.
21. Krahn AD, Manfreda J, Tate RB, Mathewson FA, Cuddy TE. The natural history of atrial fibrillation: incidence, risk factors, and prognosis in the Manitoba Follow-Up Study. Am J Med 1995;98:476-84.

For peer review only - http://bmjopen.bmj.com/site/about/guidelines.xhtml

22. Psaty BM, Manolio TA, Kuller LH, et al. Incidence of and risk factors for atrial fibrillation in older adults. Circulation 1997;96:2455-61.
23. Benjamin EJ, Levy D, Vaziri SM, D'Agostino RB, Belanger AJ, Wolf PA. Independent risk factors for atrial fibrillation in a population-based cohort. The Framingham Heart Study. JAMA 1994;271:840-4.
24. Lowres N, Olivier J, Chao TF, et al. Estimated stroke risk, yield, and number needed to screen for atrial fibrillation detected through single time screening: a multicountry patient-level meta-analysis of 141,220 screened individuals. PLoS Med 2019;16:e1002903.
25. QOF database. (Accessed 21/02/2020, at https://www.gpcontract.co.uk/.)
26. Connolly SJ, Ezekowitz MD, Yusuf S, et al. Dabigatran versus warfarin in patients with atrial fibrillation. N Engl J Med 2009;361:1139-51.
27. Robson J, Dostal I, Mathur R, et al. Improving anticoagulation in atrial fibrillation: observational study in three primary care trusts. Br J Gen Pract 2014;64:e275-e81.
28. Senoo K, Lau YC, Lip GY. Updated NICE guideline: management of atrial fibrillation (2014). Expert Rev Cardiovasc Ther 2014;12:1037-40.
29. Wilke T, Bauer S, Mueller S, Kohlmann T, Bauersachs R. Patient Preferences for Oral Anticoagulation Therapy in Atrial Fibrillation: A Systematic Literature Review. Patient 2017;10:17-37.
30. Hirst Y, Stoffel S, Baio G, McGregor L, von Wagner C. Uptake of the English Bowel (Colorectal) Cancer Screening Programme: an update 5 years after the full roll-out. Eur J Cancer 2018;103:267-73.
31. General Practice Workforce. 2020. (Accessed 06/06/2020, at https://digital.nhs.uk/data-and-information/publications/statistical/general-and-personal-medical-services/final-31-december-2019.)
32. Khurshid S, Healey JS, McIntyre WF, Lubitz SA. Population-Based Screening for Atrial Fibrillation. Circ Res 2020;127:143-54.
33. Diederichsen SZ, Haugan KJ, Brandes A, et al. Natural History of Subclinical Atrial Fibrillation Detected by Implanted Loop Recorders. J Am Coll Cardiol 2019;74:2771-81.
34. Lown M, Garrard J, Irving G, Edwards D, Hobbs FR, Mant J. Should we screen for atrial fibrillation? British Journal of General Practice 2017;67:296-7.

For peer review only

For peer review only - http://bmjopen.bmj.com/site/about/guidelines.xhtml

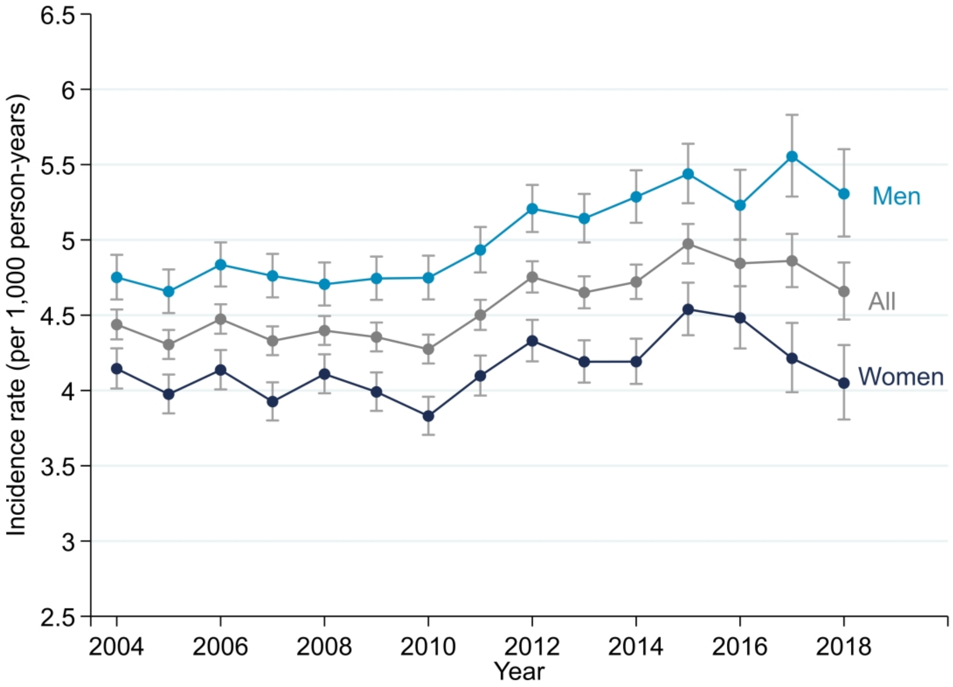

Figure 1: Modelled incidence of recorded AF over time.

1411x1026mm (72 x 72 DPI)

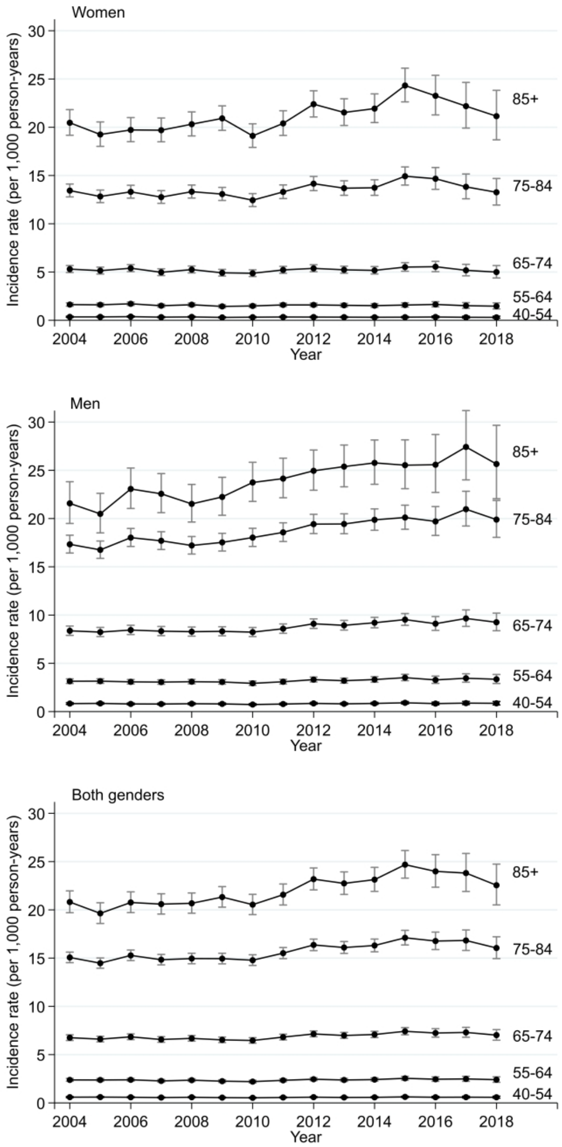

Figure 2: Modelled incidence of recorded AF over time stratified by age group and gender.

1411x2822mm (72 x 72 DPI)

Appendices

Appendix 1: Number of practices contributing data to CPRD GOLD during study period. Figures are mid-year counts.

	2004	2006	2008	2010	2012	2014	2016	2018
UK practices	680	708	717	703	672	599	448	367
English practices	451	460	462	443	411	335	185	110
Scottish, Welsh and Northern Irish practices	229	248	255	260	261	264	263	257

Appendix 2: List of codes used to define Atrial Fibrillation. The presence of these codes in the patient record, in the clinical and referral tables, was considered as evidence of diagnosis.

Medcode	Readcode	Readterm
1664	G573000	Atrial fibrillation
2212	G573.00	Atrial fibrillation and flutter
1268	G573200	Paroxysmal atrial fibrillation
1757	G573100	Atrial flutter
23437	G573z00	Atrial fibrillation and flutter NOS
96076	G573500	Persistent atrial fibrillation
96277	G573400	Permanent atrial fibrillation
35127	G573300	Non-rheumatic atrial fibrillation

For peer review only

For peer review only - http://bmjopen.bmj.com/site/about/guidelines.xhtml

Appendix 3: Flowchart for cohort - incident cases of AF from English practices.

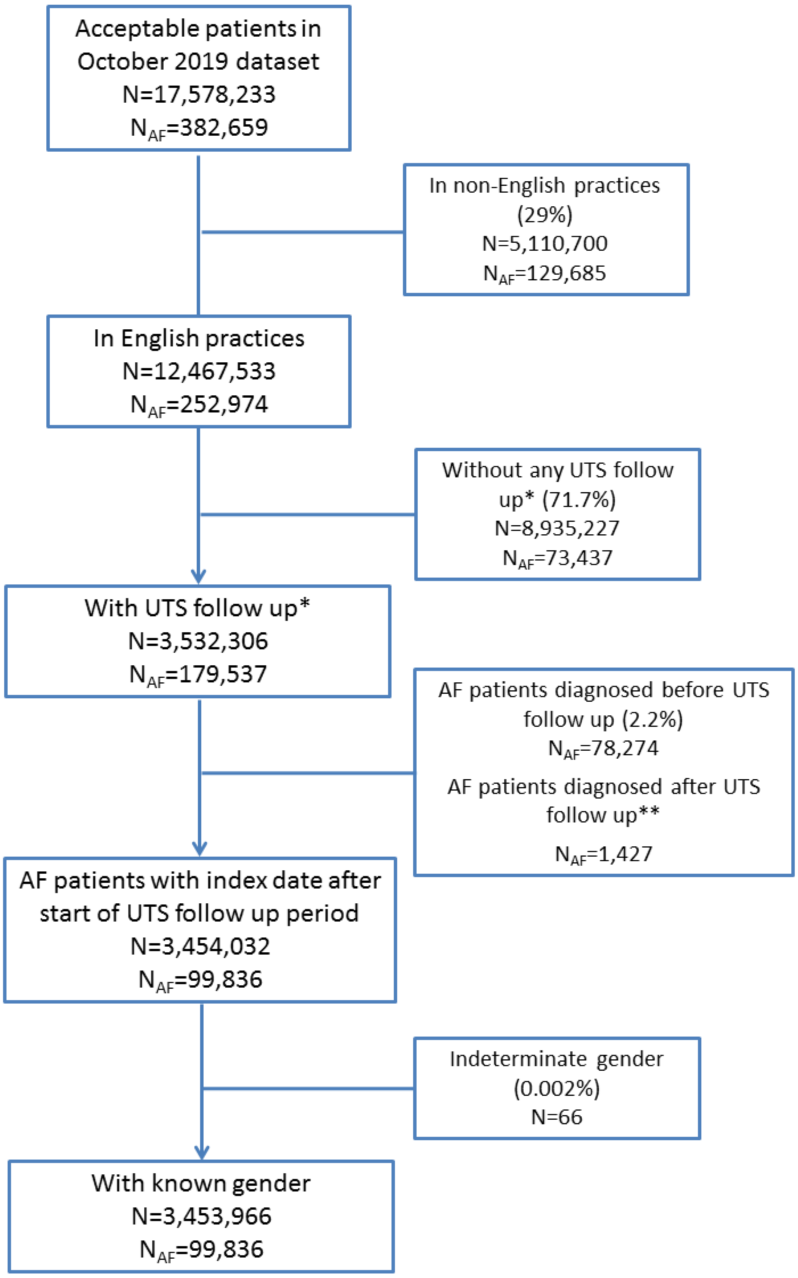

Notes: Each step shows the total number of patients in the denominator (N) and the sub-sample with AF in the numerator (N_{AF}). *"Up-to-standard" (UTS) follow up as defined in the methods section. ** N patients to be excluded from numerator but will contribute to denominator.

For peer review only - http://bmjopen.bmj.com/site/about/guidelines.xhtml

Appendix 4: Observed and modelled incidence rates (per 1,000 person-years) for recorded AF during the period 2004-2018– stratified by calendar year and gender.

Observed rates						
	Both genders		Women		Men	
Year	Rate	95% CI	Rate	95% CI	Rate	95% CI
2004	4.46	4.36-4.56	4.23	4.10-4.37	4.71	4.57-4.86
2005	4.31	4.21-4.40	4.03	3.90-4.16	4.61	4.46-4.75
2006	4.46	4.36-4.56	4.17	4.05-4.31	4.77	4.62-4.91
2007	4.31	4.22-4.41	3.95	3.83-4.08	4.69	4.55-4.84
2008	4.37	4.28-4.47	4.11	3.99-4.24	4.64	4.51-4.79
2009	4.32	4.23-4.42	3.98	3.85-4.10	4.69	4.55-4.84
2010	4.24	4.15-4.34	3.81	3.68-3.94	4.71	4.57-4.86
2011	4.49	4.39-4.59	4.07	3.94-4.21	4.93	4.78-5.08
2012	4.77	4.67-4.87	4.31	4.18-4.45	5.26	5.10-5.41
2013	4.69	4.58-4.80	4.17	4.04-4.32	5.24	5.08-5.41
2014	4.80	4.69-4.92	4.21	4.06-4.36	5.44	5.27-5.62
2015	5.07	4.94-5.20	4.55	4.38-4.73	5.62	5.42-5.82
2016	4.89	4.74-5.05	4.44	4.24-4.65	5.38	5.15-5.61
2017	4.77	4.60-4.95	4.02	3.81-4.25	5.56	5.30-5.84
2018	4.58	4.40-4.78	3.86	3.63-4.11	5.34	5.06-5.64
Modelled rates[(1)]						
	Both genders		Women		Men	
	Rate	95% CI	Rate	95% CI	Rate	95% CI
2004	4.44	4.34-4.54	4.14	4.01-4.28	4.75	4.60-4.90
2005	4.30	4.21-4.40	3.98	3.85-4.11	4.66	4.51-4.80
2006	4.47	4.38-4.57	4.14	4.01-4.27	4.84	4.69-4.98
2007	4.33	4.23-4.43	3.93	3.80-4.05	4.76	4.62-4.91
2008	4.40	4.30-4.49	4.11	3.98-4.24	4.71	4.56-4.85
2009	4.35	4.26-4.45	3.99	3.86-4.12	4.74	4.60-4.89
2010	4.27	4.18-4.37	3.83	3.71-3.96	4.75	4.60-4.90
2011	4.50	4.40-4.60	4.10	3.97-4.23	4.93	4.78-5.09
2012	4.75	4.65-4.86	4.33	4.19-4.47	5.21	5.05-5.37
2013	4.65	4.55-4.76	4.19	4.05-4.33	5.14	4.98-5.31
2014	4.72	4.61-4.84	4.19	4.04-4.34	5.29	5.11-5.46
2015	4.97	4.84-5.11	4.54	4.37-4.72	5.44	5.24-5.64
2016	4.84	4.69-5.00	4.48	4.28-4.69	5.23	5.00-5.47
2017	4.86	4.69-5.04	4.21	3.99-4.45	5.55	5.29-5.83
2018	4.66	4.47-4.85	4.05	3.81-4.30	5.31	5.02-5.60

(1) Modelled rates take into account the effect of changes in age-gender case mix over time.

For peer review only - http://bmjopen.bmj.com/site/about/guidelines.xhtml

Appendix 5: Observed and modelled incidence rates (per 1,000 person-years) for recorded AF during the period 2004-2018 – stratified by calendar year, age and gender.

Both genders – Observed rates										
	40-54		55-64		65-74		75-84		85+	
Year	Rate	95% CI	Rate	95% CI	Rate	95% CI	Rate	95% CI	Rate	95% CI
2004	0.56	0.51-0.62	2.33	2.19-2.48	6.99	6.69-7.29	15.1	14.6-15.7	20.0	19.0-21.2
2005	0.62	0.57-0.68	2.21	2.08-2.35	6.83	6.54-7.13	14.6	14.0-15.1	19.1	18.0-20.1
2006	0.54	0.49-0.59	2.43	2.29-2.58	6.98	6.69-7.28	15.4	14.9-16.0	20.0	19.0-21.1
2007	0.55	0.50-0.60	2.30	2.16-2.44	6.70	6.42-7.00	14.4	13.9-15.0	21.0	20.0-22.1
2008	0.54	0.49-0.59	2.41	2.28-2.56	6.86	6.57-7.15	14.8	14.3-15.4	20.4	19.4-21.5
2009	0.55	0.50-0.61	2.28	2.14-2.42	6.60	6.32-6.89	14.6	14.0-15.1	21.9	20.9-23.0
2010	0.51	0.46-0.56	2.24	2.11-2.39	6.49	6.21-6.79	14.8	14.3-15.4	20.4	19.4-21.5
2011	0.57	0.52-0.62	2.42	2.27-2.57	6.65	6.37-6.95	15.5	14.9-16.1	21.9	20.9-23.0
2012	0.57	0.52-0.63	2.42	2.27-2.58	7.27	6.97-7.58	16.5	15.9-17.1	22.8	21.7-23.9
2013	0.62	0.56-0.68	2.35	2.19-2.51	6.74	6.45-7.04	16.3	15.7-16.9	23.1	21.9-24.3
2014	0.62	0.56-0.69	2.38	2.21-2.55	6.86	6.55-7.19	16.7	16.0-17.4	23.2	22.0-24.4
2015	0.68	0.61-0.75	2.62	2.43-2.83	6.86	6.51-7.22	17.7	17.0-18.5	24.9	23.5-26.3
2016	0.64	0.56-0.73	2.41	2.20-2.65	7.18	6.76-7.63	16.5	15.6-17.4	24.9	23.2-26.6
2017	0.62	0.53-0.72	2.57	2.32-2.85	7.10	6.62-7.62	16.7	15.7-17.8	24.8	22.9-26.9
2018	0.63	0.54-0.75	2.37	2.11-2.66	6.97	6.45-7.53	15.7	14.7-16.9	23.7	21.7-26.0
**Women – Observed rates**										
	40-54		55-64		65-74		75-84		85+	
Year	Rate	95% CI	Rate	95% CI	Rate	95% CI	Rate	95% CI	Rate	95% CI
2004	0.293	0.243-0.354	1.52	1.36-1.69	5.61	5.25-5.99	13.9	13.2-14.6	19.1	17.9-20.5
2005	0.369	0.313-0.435	1.40	1.26-1.57	5.38	5.03-5.75	13.1	12.4-13.7	18.6	17.4-19.9
2006	0.319	0.267-0.380	1.64	1.48-1.81	5.70	5.34-6.09	13.6	13.0-14.3	18.7	17.6-20.0
2007	0.314	0.263-0.375	1.52	1.37-1.69	5.00	4.66-5.36	12.7	12.1-13.4	19.7	18.5-21.0
2008	0.293	0.245-0.352	1.61	1.46-1.79	5.45	5.10-5.82	13.5	12.8-14.2	19.7	18.5-21.0
2009	0.286	0.238-0.344	1.42	1.27-1.58	5.04	4.71-5.40	13.0	12.4-13.7	20.9	19.6-22.2
2010	0.283	0.234-0.341	1.50	1.35-1.67	4.87	4.54-5.22	12.8	12.2-13.5	18.4	17.3-19.7
2011	0.298	0.248-0.359	1.70	1.54-1.89	5.18	4.84-5.55	13.3	12.7-14.1	20.3	19.0-21.6
2012	0.306	0.255-0.368	1.49	1.33-1.67	5.56	5.21-5.94	14.6	13.8-15.3	21.5	20.2-22.9
2013	0.394	0.333-0.466	1.39	1.23-1.58	5.10	4.75-5.47	14.0	13.3-14.8	21.4	20.1-22.8
2014	0.340	0.280-0.412	1.48	1.30-1.68	4.79	4.43-5.17	14.8	14.0-15.6	21.0	19.6-22.4
2015	0.330	0.265-0.411	1.59	1.38-1.82	5.11	4.71-5.55	16.1	15.1-17.1	23.2	21.6-25.0
2016	0.339	0.262-0.439	1.61	1.37-1.89	5.73	5.22-6.29	14.3	13.2-15.4	23.6	21.7-25.8
2017	0.398	0.304-0.521	1.47	1.21-1.78	4.91	4.37-5.52	13.5	12.3-14.8	23.2	20.9-25.7
2018	0.309	0.221-0.433	1.56	1.28-1.91	4.83	4.25-5.49	13.2	11.9-14.6	21.5	19.1-24.2

For peer review only - http://bmjopen.bmj.com/site/about/guidelines.xhtml

Men – Observed rates										
	40-54		55-64		65-74		75-84		85+	
Year	Rate	95% CI	Rate	95% CI	Rate	95% CI	Rate	95% CI	Rate	95% CI
2004	0.824	0.738-0.920	3.13	2.91-3.38	8.52	8.06-9.02	17.0	16.1-17.9	22.2	20.2-24.5
2005	0.870	0.783-0.967	3.01	2.79-3.25	8.44	7.98-8.93	16.8	15.9-17.7	20.1	18.2-22.2
2006	0.750	0.669-0.839	3.22	2.99-3.46	8.40	7.93-8.88	17.9	17.0-18.9	23.1	21.1-25.3
2007	0.773	0.692-0.863	3.08	2.86-3.31	8.58	8.12-9.07	16.9	16.0-17.8	24.0	22.0-26.1
2008	0.770	0.689-0.859	3.21	2.99-3.45	8.41	7.95-8.89	16.7	15.9-17.6	22.1	20.2-24.1
2009	0.806	0.724-0.898	3.15	2.92-3.39	8.31	7.86-8.79	16.7	15.8-17.6	24.3	22.4-26.4
2010	0.725	0.647-0.813	2.99	2.77-3.23	8.28	7.83-8.76	17.5	16.6-18.4	24.8	22.9-27.0
2011	0.828	0.742-0.923	3.14	2.91-3.39	8.28	7.82-8.76	18.4	17.4-19.4	25.5	23.5-27.7
2012	0.834	0.748-0.931	3.36	3.12-3.63	9.15	8.67-9.66	19.1	18.2-20.1	25.4	23.4-27.6
2013	0.839	0.749-0.940	3.31	3.06-3.59	8.55	8.08-9.05	19.4	18.4-20.4	26.6	24.5-28.8
2014	0.901	0.802-1.013	3.28	3.01-3.58	9.15	8.63-9.70	19.2	18.2-20.3	27.6	25.4-30.1
2015	1.015	0.897-1.149	3.67	3.35-4.01	8.79	8.22-9.39	19.9	18.7-21.1	28.2	25.7-30.9
2016	0.927	0.795-1.082	3.22	2.87-3.60	8.79	8.12-9.52	19.4	18.0-21.0	27.3	24.4-30.5
2017	0.832	0.692-1.000	3.65	3.24-4.12	9.52	8.71-10.4	20.8	19.1-22.7	27.8	24.4-31.6
2018	0.952	0.788-1.150	3.17	2.76-3.65	9.32	8.46-10.3	19.1	17.3-21.0	27.6	24.0-31.8

Both genders – Modelled rates(1)										
	40-54		55-64		65-74		75-84		85+	
Year	Rate	95% CI	Rate	95% CI	Rate	95% CI	Rate	95% CI	Rate	95% CI
2004	0.594	0.540-0.652	2.38	2.24-2.54	6.76	6.47-7.06	15.1	14.5-15.6	20.8	19.7-22.0
2005	0.604	0.550-0.661	2.38	2.24-2.52	6.61	6.32-6.91	14.5	14.0-15.0	19.6	18.6-20.7
2006	0.588	0.536-0.644	2.39	2.25-2.54	6.85	6.56-7.15	15.3	14.7-15.8	20.8	19.7-21.9
2007	0.558	0.507-0.612	2.28	2.14-2.42	6.57	6.28-6.86	14.8	14.3-15.4	20.6	19.6-21.7
2008	0.586	0.534-0.641	2.35	2.22-2.50	6.69	6.40-6.98	15.0	14.4-15.5	20.7	19.7-21.7
2009	0.550	0.500-0.604	2.25	2.12-2.40	6.53	6.25-6.82	15.0	14.4-15.5	21.3	20.3-22.4
2010	0.528	0.479-0.581	2.20	2.07-2.35	6.47	6.19-6.76	14.8	14.2-15.3	20.5	19.5-21.6
2011	0.565	0.513-0.620	2.34	2.19-2.49	6.82	6.52-7.12	15.5	14.9-16.1	21.6	20.5-22.7
2012	0.595	0.541-0.653	2.45	2.30-2.61	7.15	6.85-7.45	16.4	15.8-17.0	23.2	22.1-24.3
2013	0.570	0.515-0.628	2.38	2.22-2.54	6.99	6.69-7.30	16.1	15.5-16.7	22.7	21.6-23.9
2014	0.581	0.523-0.645	2.42	2.25-2.59	7.09	6.77-7.42	16.3	15.7-17.0	23.1	21.9-24.4
2015	0.621	0.553-0.695	2.55	2.35-2.75	7.42	7.06-7.80	17.1	16.4-17.9	24.7	23.3-26.1
2016	0.589	0.511-0.676	2.45	2.23-2.69	7.24	6.81-7.69	16.8	15.9-17.7	24.0	22.3-25.7
2017	0.595	0.506-0.694	2.48	2.23-2.76	7.30	6.81-7.83	16.8	15.8-17.9	23.8	21.9-25.8
2018	0.585	0.489-0.694	2.41	2.14-2.70	7.02	6.49-7.59	16.1	15.0-17.2	22.6	20.5-24.7

For peer review only - http://bmjopen.bmj.com/site/about/guidelines.xhtml

Women – Modelled rates[1]										
	40-54		55-64		65-74		75-84		85+	
Year	Rate	95% CI	Rate	95% CI	Rate	95% CI	Rate	95% CI	Rate	95% CI
2004	0.350	0.292-0.416	1.63	1.46-1.81	5.30	4.95-5.67	13.4	12.8-14.1	20.5	19.2-21.8
2005	0.350	0.293-0.415	1.60	1.44-1.78	5.14	4.79-5.50	12.8	12.2-13.5	19.3	18.0-20.5
2006	0.378	0.319-0.445	1.71	1.54-1.88	5.40	5.04-5.77	13.3	12.7-14.0	19.7	18.5-21.0
2007	0.322	0.268-0.383	1.51	1.35-1.68	4.97	4.63-5.33	12.8	12.1-13.4	19.7	18.5-21.0
2008	0.346	0.291-0.409	1.61	1.45-1.78	5.25	4.91-5.62	13.3	12.7-14.0	20.3	19.1-21.6
2009	0.296	0.245-0.355	1.44	1.29-1.60	4.92	4.58-5.27	13.1	12.4-13.8	20.9	19.7-22.2
2010	0.318	0.265-0.380	1.48	1.33-1.65	4.87	4.54-5.22	12.4	11.8-13.1	19.1	17.9-20.4
2011	0.340	0.284-0.404	1.59	1.42-1.77	5.22	4.87-5.59	13.3	12.6-14.0	20.4	19.1-21.7
2012	0.330	0.274-0.394	1.59	1.42-1.78	5.38	5.03-5.75	14.2	13.4-14.9	22.4	21.1-23.8
2013	0.325	0.268-0.391	1.55	1.38-1.74	5.23	4.87-5.60	13.7	12.9-14.5	21.5	20.2-23.0
2014	0.313	0.253-0.383	1.52	1.33-1.72	5.17	4.79-5.57	13.7	12.9-14.6	21.9	20.5-23.5
2015	0.318	0.251-0.397	1.58	1.37-1.81	5.51	5.08-5.97	14.9	14.0-15.9	24.3	22.6-26.1
2016	0.339	0.258-0.439	1.64	1.39-1.92	5.55	5.04-6.10	14.7	13.6-15.8	23.3	21.3-25.4
2017	0.308	0.221-0.418	1.51	1.24-1.83	5.18	4.60-5.80	13.8	12.6-15.2	22.2	19.9-24.6
2018	0.300	0.207-0.422	1.46	1.17-1.80	5.00	4.38-5.67	13.3	11.9-14.7	21.1	18.7-23.8

Men – Modelled rates[1]										
	40-54		55-64		65-74		75-84		85+	
Year	Rate	95% CI	Rate	95% CI	Rate	95% CI	Rate	95% CI	Rate	95% CI
2004	0.829	0.740-0.925	3.14	2.91-3.39	8.37	7.90-8.86	17.3	16.4-18.3	21.6	19.5-23.8
2005	0.847	0.759-0.943	3.15	2.92-3.40	8.24	7.77-8.72	16.8	15.9-17.7	20.5	18.5-22.6
2006	0.792	0.707-0.884	3.08	2.86-3.32	8.45	7.98-8.94	18.0	17.1-19.0	23.1	21.0-25.2
2007	0.787	0.703-0.878	3.06	2.83-3.29	8.33	7.87-8.82	17.7	16.8-18.6	22.6	20.6-24.7
2008	0.816	0.731-0.908	3.10	2.87-3.33	8.28	7.82-8.76	17.2	16.3-18.1	21.5	19.6-23.5
2009	0.797	0.713-0.888	3.07	2.84-3.30	8.31	7.85-8.79	17.5	16.6-18.5	22.2	20.3-24.3
2010	0.732	0.652-0.821	2.93	2.71-3.17	8.23	7.77-8.71	18.0	17.1-19.0	23.7	21.8-25.8
2011	0.782	0.696-0.874	3.09	2.85-3.34	8.58	8.11-9.08	18.6	17.6-19.6	24.1	22.2-26.3
2012	0.850	0.760-0.947	3.31	3.06-3.58	9.10	8.61-9.60	19.4	18.4-20.4	25.0	22.9-27.1
2013	0.806	0.715-0.905	3.20	2.94-3.47	8.94	8.44-9.45	19.4	18.4-20.5	25.4	23.3-27.6
2014	0.843	0.744-0.952	3.32	3.04-3.62	9.21	8.68-9.76	19.9	18.8-21.0	25.8	23.6-28.1
2015	0.910	0.795-1.037	3.52	3.20-3.86	9.53	8.93-10.16	20.1	18.9-21.4	25.5	23.1-28.2
2016	0.829	0.699-0.976	3.28	2.92-3.67	9.11	8.41-9.84	19.7	18.3-21.2	25.6	22.7-28.7
2017	0.876	0.725-1.048	3.46	3.04-3.92	9.65	8.82-10.53	21.0	19.2-22.8	27.4	24.0-31.2
2018	0.854	0.692-1.043	3.35	2.91-3.84	9.25	8.37-10.20	19.9	18.0-21.9	25.7	22.1-29.7

(1) Modelled rates take into account the effect of changes in the age-gender case mix over time.

For peer review only - http://bmjopen.bmj.com/site/about/guidelines.xhtml

Appendix 6: Modelled incidence of recorded AF over time by patient deprivation (most vs least deprived quintiles of the Index of Multiple Deprivation (IMD). The effect of deprivation was shown to be statistically significant (p=0.002, see also appendix 7).

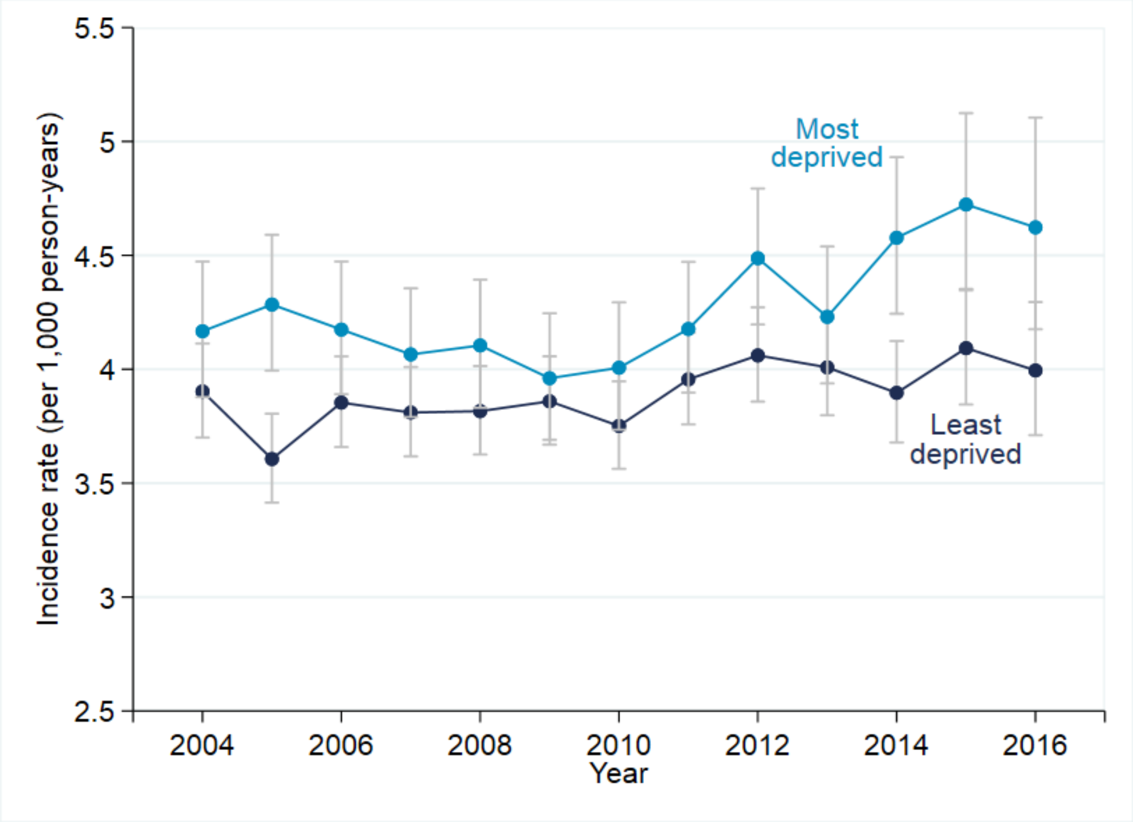

For peer review only - http://bmjopen.bmj.com/site/about/guidelines.xhtml

Appendix 7: Model results for the association between the Index of Multiple Deprivation (IMD) and incidence rates. Relative differences between deprivation categories are shown as incidence rate ratios (IRR).

	IRR (95% CI)	p
IMD (ref=1, least deprived)		0.002
2	1.06 (1.02-1.10)	
3	1.07 (1.01-1.14)	
4	1.10 (1.01-1.20)	
5, most deprived	1.17 (1.05-1.31)	
c.IMD*i.year (ref=2016)		0.182
2004	0.98 (0.95-1.01)	
2005	1.01 (0.97-1.04)	
2006	0.98 (0.95-1.02)	
2007	0.98 (0.95-1.01)	
2008	0.98 (0.95-1.02)	
2009	0.97 (0.94-1.00)	
2010	0.98 (0.95-1.01)	
2011	0.98 (0.95-1.01)	
2012	0.99 (0.96-1.02)	
2013	0.98 (0.95-1.01)	
2014	1.00 (0.97-1.04)	
2015	1.00 (0.97-1.04)	

Notes:

1) The model structure is as follows (as described in the methods section): Year + agecat + gender + IMD + i.year*c.agecat + i.year*i.gender + i.year*c.IMD + c.agecat*i.gender + i.year*c.agecat*i.gender

2) This analysis uses a subsample of patients with complete IMD during the period 2004 to 2016. It includes a total of 74,514 AF incident cases and 17.8 million person-years of observation. Due to methodological differences between the IMD stratified denominator provided by CPRD and the way we have calculated the incidence rates, the rate for all patients in this subsample is slightly lower than our main analysis rate. Therefore we prefer to show relative differences (as incidence rate ratios, IRR) instead of absolute differences in the quintiles of IMD.

For peer review only

For peer review only - http://bmjopen.bmj.com/site/about/guidelines.xhtml

Appendix 8: Observed incidence rates (per 1,000 person-years) for recorded AF during the period 2004-2018. This is an additional stratification by age (under and over 65), for comparison with other studies.

	Incidence rate	95% CI
40-64	1.21	1.19-1.23
65+	11.6	11.5-11.6
Women		
40-64	0.75	0.73-0.77
65+	10.4	10.3-10.5
Men		
40-64	1.66	1.63-1.69
65+	13.0	12.9-13.2

For peer review only

The RECORD statement – checklist of items, extended from the STROBE statement, that should be reported in observational studies using routinely collected health data.

	Item No.	STROBE items	Location in manuscript where items are reported	RECORD items	Location in manuscript where items are reported
**Title and abstract**					
	1	(a) Indicate the study's design with a commonly used term in the title or the abstract (b) Provide in the abstract an informative and balanced summary of what was done and what was found		RECORD 1.1: The type of data used should be specified in the title or abstract. When possible, the name of the databases used should be included.<br><br>RECORD 1.2: If applicable, the geographic region and timeframe within which the study took place should be reported in the title or abstract.<br><br>RECORD 1.3: If linkage between databases was conducted for the study, this should be clearly stated in the title or abstract.	Abstract –setting<br><br>Abstract – objective and participants<br><br>Abstract - Setting
**Introduction**					
Background rationale	2	Explain the scientific background and rationale for the investigation being reported	Introduction – sentences 4 to 10		
Objectives	3	State specific objectives, including any prespecified hypotheses	Last sentence of introduction		
**Methods**					
Study Design	4	Present key elements of study design early in the paper	Methods – Data source and Study cohort and disease definition		
Setting	5	Describe the setting, locations, and relevant dates, including	Methods –Study cohort and disease definition		

For peer review only

		periods of recruitment, exposure, follow-up, and data collection			
Participants	6	*(a) Cohort study* - Give the eligibility criteria, and the sources and methods of selection of participants. Describe methods of follow-up<br>*Case-control study* - Give the eligibility criteria, and the sources and methods of case ascertainment and control selection. Give the rationale for the choice of cases and controls<br>*Cross-sectional study* - Give the eligibility criteria, and the sources and methods of selection of participants<br><br>*(b) Cohort study* - For matched studies, give matching criteria and number of exposed and unexposed<br>*Case-control study* - For matched studies, give matching criteria and the number of controls per case		RECORD 6.1: The methods of study population selection (such as codes or algorithms used to identify subjects) should be listed in detail. If this is not possible, an explanation should be provided.<br><br>RECORD 6.2: Any validation studies of the codes or algorithms used to select the population should be referenced. If validation was conducted for this study and not published elsewhere, detailed methods and results should be provided.<br><br>RECORD 6.3: If the study involved linkage of databases, consider use of a flow diagram or other graphical display to demonstrate the data linkage process, including the number of individuals with linked data at each stage.	Methods - Study cohort and disease definition. Codes used to identify AF cases provided in Appendix 2.<br><br>Methods - Study cohort and disease definition references where our codelist was adapted from.<br><br>The flowchart in Appendix 3 shows how we identified patients contributing to the numerator/denominator.
Variables	7	Clearly define all outcomes, exposures, predictors, potential confounders, and effect modifiers. Give diagnostic criteria, if applicable.		RECORD 7.1: A complete list of codes and algorithms used to classify exposures, outcomes, confounders, and effect modifiers should be provided. If these cannot be reported, an explanation should be provided.	Codes used to identify AF cases are provided in Appendix 2.
Data sources/ measurement	8	For each variable of interest, give sources of data and details of methods of assessment (measurement).	In Methods – Data analysis we provide a detailed explanation for how eligible time of follow up and AF		

For peer review only

		Describe comparability of assessment methods if there is more than one group	cases were determined – paragraphs 1 and 2, respectively.		
Bias	9	Describe any efforts to address potential sources of bias	In Methods – Data analysis paragraph 6 we explain the approach taken to address a potential source of bias.		
Study size	10	Explain how the study size was arrived at	The flowchart in Appendix 3 shows how we arrived at the final numbers of patients contributing to the numerator/denominator.		
Quantitative variables	11	Explain how quantitative variables were handled in the analyses. If applicable, describe which groupings were chosen, and why	In Methods – Data analysis paragraph 4 we explain how we categorized age.		
Statistical methods	12	(a) Describe all statistical methods, including those used to control for confounding<br>(b) Describe any methods used to examine subgroups and interactions<br>(c) Explain how missing data were addressed<br>(d) *Cohort study* - If applicable, explain how loss to follow-up was addressed<br>*Case-control study* - If applicable, explain how matching of cases and controls was addressed	(a) We used stratification and modelling which were described in paragraphs 5 and 6 of Methods – Data analysis.<br>(b) The modelling approach included interaction terms described in paragraph 6 of Data analysis.<br>(c) This study focuses on recorded		

For peer review only

		Cross-sectional study - If applicable, describe analytical methods taking account of sampling strategy<br>(e) Describe any sensitivity analyses	AF so it doesn't include patients whose diagnosis is not recorded in their medical records – this is clarified in the Strengths & Limitation section, sentence 2.<br>(d) only time during eligible follow-up is included in the analysis; this is described in paragraph 1 of Data Analysis.<br>(e) Not applicable		
Data access and cleaning methods		..		RECORD 12.1: Authors should describe the extent to which the investigators had access to the database population used to create the study population.<br><br>RECORD 12.2: Authors should provide information on the data cleaning methods used in the study.	The information we used to identify cases is explained in methods-data analysis paragraphs 1 and 2.<br>This is an incidence study that did not involve a lot of data cleaning. We explain how we handled events after death in methods-data analysis paragraph 2.

For peer review only

Linkage		..		RECORD 12.3: State whether the study included person-level, institutional-level, or other data linkage across two or more databases. The methods of linkage and methods of linkage quality evaluation should be provided.	Included in Methods – Data analysis paragraph 7. A link to details on the IMD-linkage is available in the data sharing statement.
**Results**					
Participants	13	(a) Report the numbers of individuals at each stage of the study (*e.g.*, numbers potentially eligible, examined for eligibility, confirmed eligible, included in the study, completing follow-up, and analysed)<br>(b) Give reasons for non-participation at each stage.<br>(c) Consider use of a flow diagram		RECORD 13.1: Describe in detail the selection of the persons included in the study (*i.e.,* study population selection) including filtering based on data quality, data availability and linkage. The selection of included persons can be described in the text and/or by means of the study flow diagram.	Flowchart for selected patients shown in Appendix 3. Final numbers reported in Results paragraph 1.
Descriptive data	14	(a) Give characteristics of study participants (*e.g.*, demographic, clinical, social) and information on exposures and potential confounders<br>(b) Indicate the number of participants with missing data for each variable of interest<br>(c) *Cohort study* - summarise follow-up time (*e.g.*, average and total amount)	(a)Patient characteristics reported in results section paragraph 1 and Table 1.<br>(b) Participants excluded because of missing data for gender are shown in Appendix 3.<br>(c) Total follow up time reported in the first sentence of Results section.		
Outcome data	15	*Cohort study* - Report numbers of outcome events or summary measures over time	Number of new AF cases in the study period is reported in		

For peer review only

		Case-control study - Report numbers in each exposure category, or summary measures of exposure<br>*Cross-sectional study* - Report numbers of outcome events or summary measures	the first sentence of Results section.		
Main results	16	(a) Give unadjusted estimates and, if applicable, confounder-adjusted estimates and their precision (e.g., 95% confidence interval). Make clear which confounders were adjusted for and why they were included<br>(b) Report category boundaries when continuous variables were categorized<br>(c) If relevant, consider translating estimates of relative risk into absolute risk for a meaningful time period	(a) Observed rates stratified by age and gender are shown in Table 2. Modelled (adjusted) rates are shown in Figures 1 and 2 and appendix 6. Both observed and modelled rates are included in Appendices 4 and 5.<br>(b) Not applicable<br>(c) Our main analysis presents absolute risk estimates as incidence rates.		
Other analyses	17	Report other analyses done—e.g., analyses of subgroups and interactions, and sensitivity analyses	One additional analysis including deprivation is included as Appendix 6 and 7.		
**Discussion**					
Key results	18	Summarise key results with reference to study objectives	Shown in Discussion - Summary		
Limitations	19	Discuss limitations of the study, taking into account sources of potential bias or imprecision. Discuss both direction and magnitude of any potential bias		RECORD 19.1: Discuss the implications of using data that were not created or collected to answer the specific research question(s). Include discussion of misclassification bias, unmeasured confounding, missing	Included in Discussion - Strengths and limitations

For peer review only

				data, and changing eligibility over time, as they pertain to the study being reported.	
Interpretation	20	Give a cautious overall interpretation of results considering objectives, limitations, multiplicity of analyses, results from similar studies, and other relevant evidence	Included in Discussion – Implication for research, policy and practice paragraph 1.		
Generalisability	21	Discuss the generalisability (external validity) of the study results	Included in Discussion - comparison with existing literature.		
**Other Information**					
Funding	22	Give the source of funding and the role of the funders for the present study and, if applicable, for the original study on which the present article is based	Included in Funding statement.		
Accessibility of protocol, raw data, and programming code		..		RECORD 22.1: Authors should provide information on how to access any supplemental information such as the study protocol, raw data, or programming code.	Included in the Data sharing statement.

*Reference: Benchimol EI, Smeeth L, Guttmann A, Harron K, Moher D, Petersen I, Sørensen HT, von Elm E, Langan SM, the RECORD Working Committee. The REporting of studies Conducted using Observational Routinely-collected health Data (RECORD) Statement. *PLoS Medicine* 2015; in press.

*Checklist is protected under Creative Commons Attribution (CC BY) license.

For peer review only
